# Cardiovascular adverse effects of immunotherapy in cancer: insights and implications

**DOI:** 10.3389/fonc.2025.1601808

**Published:** 2025-06-18

**Authors:** Haiping Du, Jie Wang, Zhen Wang

**Affiliations:** ^1^ Department of Cardiology, Yantaishan Hospital, Yantai, Shandong, China; ^2^ Department of Cardiac Care Unit, Yantaishan Hospital, Yantai, Shandong, China

**Keywords:** cardiotoxicity, immunotherapy, immune checkpoint inhibitors, CAR T-cell therapy, cancer vaccines, cytokine-mediated inflammation, cardio-oncology

## Abstract

Immunotherapy has revolutionized cancer treatment, offering novel therapeutic strategies such as immune checkpoint inhibitors (ICIs), chimeric antigen receptor (CAR) T-cell therapy, and cancer vaccines. However, these modalities are associated with varying cardiovascular toxicities that may affect treatment continuation and patient outcomes. Cardiovascular complications from ICIs, such as myocarditis (incidence 0.04–1.14%, mortality 25–50%), arrhythmias, heart failure, and thromboembolic events, are primarily mediated by autoreactive T-cell activation and immune-related inflammation. CTLA-4 and PD-1/PD-L1 blockade disrupts immune homeostasis, leading to direct myocardial infiltration and cytokine-mediated damage. Up to 26% of patients receiving CAR T-cell therapy develop cardiovascular events, often secondary to cytokine release syndrome (CRS). Excessive release of pro-inflammatory cytokines (e.g., IL-6, IFN-γ) leads to endothelial dysfunction, hypotension, myocardial depression, arrhythmias, and acute coronary syndromes. Rare cases of myocarditis and arrhythmias have been reported following mRNA vaccine administration, particularly in younger males. Proposed mechanisms include innate immune activation via Toll-like receptors, leading to cytokine release and myocardial inflammation. Dendritic cell vaccines show lower cardiovascular toxicity, likely due to their localized and cell-specific immune activation. This review provides a comprehensive evaluation of cardiovascular adverse events across immunotherapy classes. It underscores the importance of early detection through biomarkers, risk stratification, and multidisciplinary cardio-oncology collaboration. Future research should aim to refine immunotherapy protocols to minimize cardiotoxic risks while preserving anti-tumor efficacy.

## 1 Introduction

Over the last few decades, extensive oncology research has improved our understanding of the molecular pathways driving tumor cell proliferation, therapeutic resistance, metastatic propagation, and immune evasion (1–3). As a result, several new compounds have been developed, and some potential medicinal medicines are now being evaluated for regulatory approval. Among these advances, immunotherapy has emerged as one of the most promising treatments for treating systemic cancer (4).

An important part of oncologic treatment, in addition to therapeutic success, is dealing with the side effects of anticancer drugs. While drug-induced toxicities can affect many organ systems, cardiovascular problems are among the most serious because they have a significant impact on patient prognosis and overall quality of life. The cardiotoxic effects of therapies such as anthracyclines and radiation therapy are widely known and extensively researched in the literature (5). The immune systems of cancer patients are significantly compromised, as tumor cells employ various mechanisms to evade detection by dendritic cells, thus hindering effective immune surveillance (6). In cancer patients, the immune system’s ability to identify and eliminate malignant cells is greatly weakened. Immunotherapy aims to retrain the dysregulated immune response and restore its anticancer activity. Dendritic cell-based cancer vaccines and adoptive cell transfer methods are examples of active immunotherapy (4, 7, 8). These therapeutic approaches involve the ex vivo manipulation of the patient’s dendritic cells (DCs) or T cells, followed by their reinfusion to enhance antitumor immune responses (8, 9). Passive immunotherapy encompasses a diverse range of therapeutic modalities, including monoclonal antibodies, immune checkpoint inhibitors, cytokines, and bispecific T-cell engagers. These biologics are pivotal role in modulating key physiological mechanisms that influence tumor progression, angiogenic processes, and T-cell-mediated immune responses (4). Both approaches have yielded encouraging outcomes across various malignancies. A comprehensive understanding of the underlying mechanisms contributing to their distinct cardiotoxic profiles is essential for identifying high-risk individuals and optimizing oncologic treatment strategies to mitigate cardiovascular complications (10, 11). With an emphasis on CAR T-cell therapy, immune checkpoint inhibitors, and other immunotherapeutic strategies, this review article attempts to present a thorough summary of the cardiovascular side effects connected to immunotherapy in the treatment of cancer. The goal is to elucidate the underlying mechanisms, identify risk factors, and discuss management strategies to mitigate these complications, ultimately improving patient safety and therapeutic outcomes in cancer immunotherapy.

## 2 Importance of immunotherapy

After heart disease, cancer is the second most common cause of mortality globally. According to recent data from GLOBOCAN 2020, cancers killed around 10 million people worldwide in 2020 alone (12). Breast cancer remains the most commonly diagnosed malignancy globally, with approximately 2 million new cases reported annually. It is followed by cancers of the stomach, non-melanoma skin, prostate, lung, and colorectal regions. In addition, projections indicate a substantial rise in the global elderly population in the coming years. This demographic shift is expected to increase cancer susceptibility, as aging is often accompanied by a decline in physiological resilience and overall health, making older individuals more vulnerable to malignancies (13).

Despite the unavoidable side effects and probable limits of cancer medicines, considerable technical advances in cancer treatment have been made during the last century. These discoveries have significantly revolutionized the landscape of cancer, although issues in treatment effectiveness and side effects continue (14). In practice, patients diagnosed at an early stage of cancer demonstrate a significantly higher likelihood of overall survival and benefit from more cost-effective treatment options compared to those diagnosed at more advanced stages of the disease. Early detection not only enhances survival outcomes but also reduces the financial burden of treatment (15). The primary goal of any therapeutic regimen is to eradicate cancer and extend the patient’s life by inhibiting or halting the proliferation of cancerous cells. However, the approach to cancer treatment can differ based on the timing of diagnosis, with early detection offering distinct advantages over late-stage diagnosis, which may involve the consideration of metastasis.

**Figure 1:**
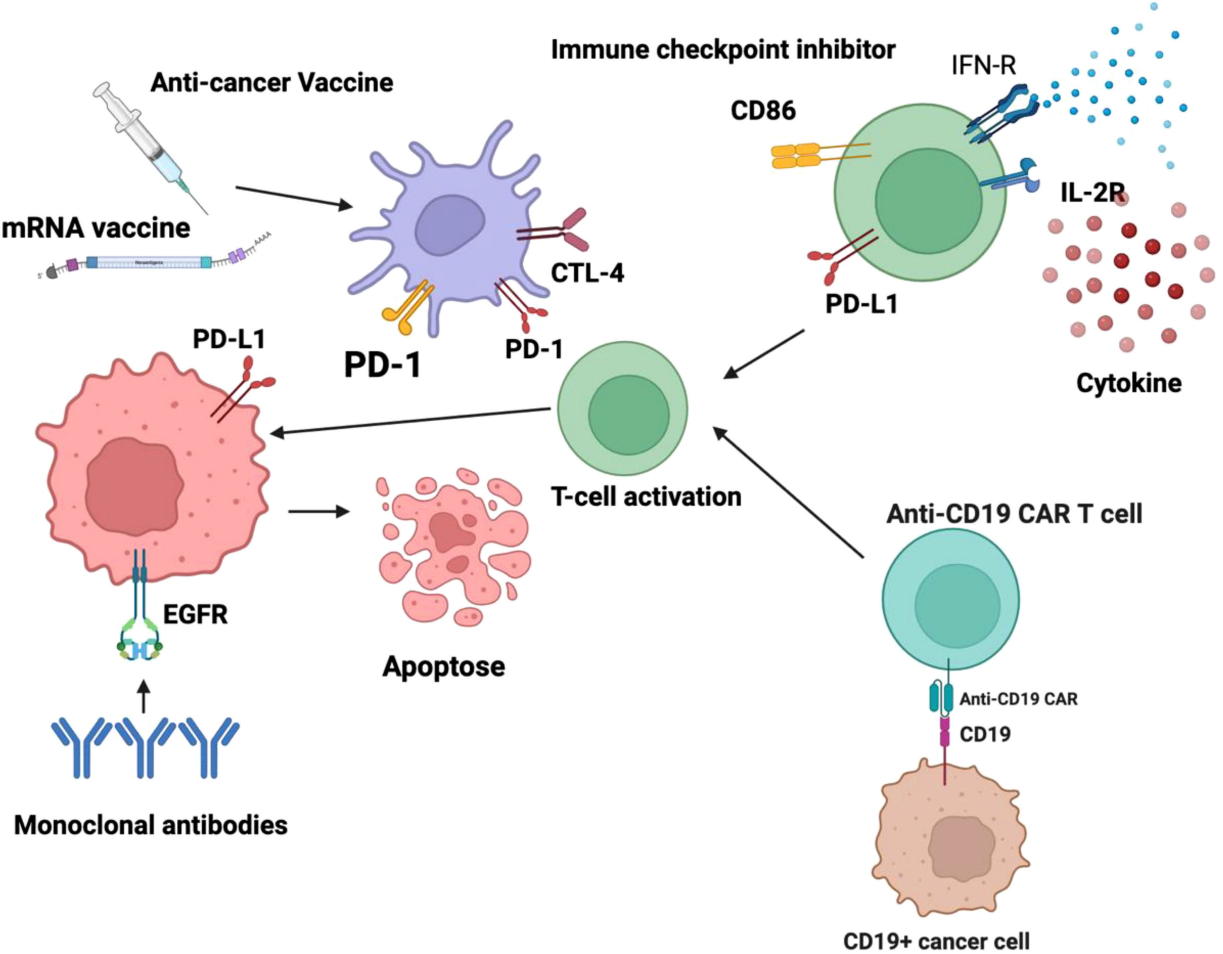
Active immunotherapy encompasses strategies designed to stimulate the body’s immune system to recognize and eliminate malignant cells. Among these approaches, dendritic cell-based cancer vaccines play a crucial role by harnessing antigen-presenting cells to initiate a robust adaptive immune response, primarily through the activation of T lymphocytes. Additionally, chimeric antigen receptor (CAR) T-cell therapy represents a sophisticated form of adoptive cellular immunotherapy, wherein T cells are genetically modified to express engineered receptors targeting specific tumor-associated antigens, thereby enhancing their cytotoxic potential. These modalities aim to induce a sustained and highly specific anti-tumor immune response, ultimately improving patient outcomes. Conversely, passive immunotherapy involves the administration of exogenous immune components that directly enhance the body’s ability to combat cancer cells without eliciting a *de novo* immune response. This category includes monoclonal antibodies, which are engineered to target specific tumor antigens, facilitating immune-mediated destruction through mechanisms such as antibody-dependent cellular cytotoxicity (ADCC) and complement activation. Checkpoint inhibitors, another class of passive immunotherapy, function by blocking immune checkpoint pathways such as PD-1/PD-L1 and CTLA-4 that cancer cells exploit to evade immune detection, thereby restoring T-cell activity against tumors. Additionally, cytokine-based therapies leverage immune-stimulatory molecules, such as interleukins and interferons, to modulate the tumor microenvironment and enhance immune effector functions. Collectively, these immunotherapeutic strategies represent significant advancements in oncology, offering targeted and personalized treatment options for various malignancies.

Chemotherapy, radiation therapy, and surgery have long been regarded as the three primary pillars of cancer treatment. Because of the effectiveness of immunotherapy, either alone or in conjunction with other cancer treatments, immunotherapy has become the fourth essential pillar in the fight against the illness (15). Unlike conventional cancer therapies, immunotherapy uses the body’s immune system to recognize and target cancer cells, providing a more organic way to manage the course of the illness. Even while traditional therapies like radiation, chemotherapy, and surgery have been successful in controlling primary tumors, cancer recurrence is still a serious worry and is frequently caused by metastases or leftover cancerous cells (16). Immunotherapy, which uses immune checkpoint inhibitors (ICIs), chimeric antigen receptor (CAR) T-cell therapy, and cancer vaccines to boost the body’s immune response against cancerous cells, therefore becomes a viable alternative or supplemental approach to cancer treatment (**Figure 1**) (17). As of 2023, ICIs such as pembrolizumab, nivolumab, and atezolizumab have been approved by the FDA and EMA for over 20 malignancies, including non-small cell lung cancer (NSCLC), melanoma, renal cell carcinoma, and head and neck squamous cell carcinoma (18, 19). Furthermore, the American Society of Clinical Oncology (ASCO) and the National Comprehensive Cancer Network (NCCN) now recommend ICIs as first-line or adjuvant therapies in multiple stage III and IV cancers. For instance, in metastatic melanoma, the introduction of ipilimumab and nivolumab has increased 5-year survival rates from under 10% to over 50% in select populations (20). In non-small cell lung cancer demonstrated that pembrolizumab led to a median overall survival of 26.3 months versus 13.4 months with chemotherapy alone in patients with PD-L1 ≥50% (21). The increasing number of clinical trials incorporating immunotherapy, accounting for approximately 43% of all oncology trials registered on ClinicalTrials.gov as of 2022 further underscores its widespread integration and growing significance. This data-driven incorporation into treatment standards, coupled with the transformative survival benefits in previously refractory cancers, underscores immunotherapy’s position as a cornerstone of modern oncology. **Table 1** summarizes the cardiovascular side effects associated with different immunotherapy modalities.

**Table 1:** The immunotherapy treatments that have been associated with cardiovascular side effects.

	Cardiovascular side effect	Reference
CAR T-cell	Hypotension QT prolongation ST segment changes Sinus tachycardia Atrial fibrillation Left ventricular systolic dysfunction Troponin elevation Cardiac arrest	(37)
Immune checkpoint inhibitor	Myocarditis Cardiomyopathy Pericarditis Arrhythmias	(250)
mRNA vaccine	Myopericarditis Myocarditis Hypotension Hypertension Arrhythmia Cardiogenic shock Stroke Myocardial Infarction/STEMI Intracranial hemorrhage Thrombosis	(199)
Monoclonal antibody	Cardiomyopathy Ventricular dysfunction Arrhythmias Arrests Acute coronary syndromes	(251)

As immunotherapy becomes increasingly integrated into frontline and adjuvant cancer treatment regimens, its associated cardiovascular toxicities are gaining attention as critical complications with real-world implications. Although considered rare, immune checkpoint inhibitor-related myocarditis occurs in approximately 0.04% to 1.14% of treated patients and is associated with a high case fatality rate, ranging from 25% to 50%, substantially higher than many other immune-related adverse events (22). Additionally, combination ICI therapies have been shown to increase both the incidence and severity of cardiovascular events, including pericarditis, arrhythmias, and heart failure. Similarly, CAR T-cell therapy is associated with cardiovascular complications in up to 26% of patients, primarily driven by cytokine release syndrome (CRS) (23). In severe cases, these toxicities can result in acute heart failure, arrhythmias, and even cardiogenic shock, particularly in vulnerable patients. Despite these significant risks, standardized cardiovascular monitoring protocols remain lacking in most oncology guidelines, and many clinical trials continue to underreport cardiovascular endpoints. This underscores a pressing need for dedicated cardio-oncology research to better define risk factors, identify early biomarkers, and develop evidence-based surveillance and mitigation strategies. As the use of immunotherapy expands to earlier-stage and more diverse patient populations, understanding and managing its cardiovascular implications is essential for optimizing long-term outcomes and maintaining quality of life (24).

## 3 Adverse effect of immunotherapy related to Cardiovascular

### 3.1 CAR T-Cell

#### 3.1.1 Clinical evidence

Clinical studies demonstrating recent advancements in immune-based therapies have demonstrated promise, providing cancer patients with the possibility of permanent remission and, in some cases, complete disease elimination (25, 26). In the continuous fight against cancer, CAR-engineered T cell adoptive immunotherapy is becoming more and more well-known as a highly targeted and personalized immune-based therapeutic approach (27, 28). CARs are artificially created fusion proteins that are purposefully made to recognize antigens linked to tumors. This helps to activate T cells and encourages the targeted destruction of cancerous cells (29). The foundational development of CAR constructs for cancer therapy began in the late 1980s and early 1990s. However, it is only in recent years that these technologies have achieved notable clinical breakthroughs especially in the management of hematologic malignancies, where CAR-based therapies have demonstrated remarkable efficacy, particularly among patients with leukemia and lymphoma (30–36).

**Figure 2:**
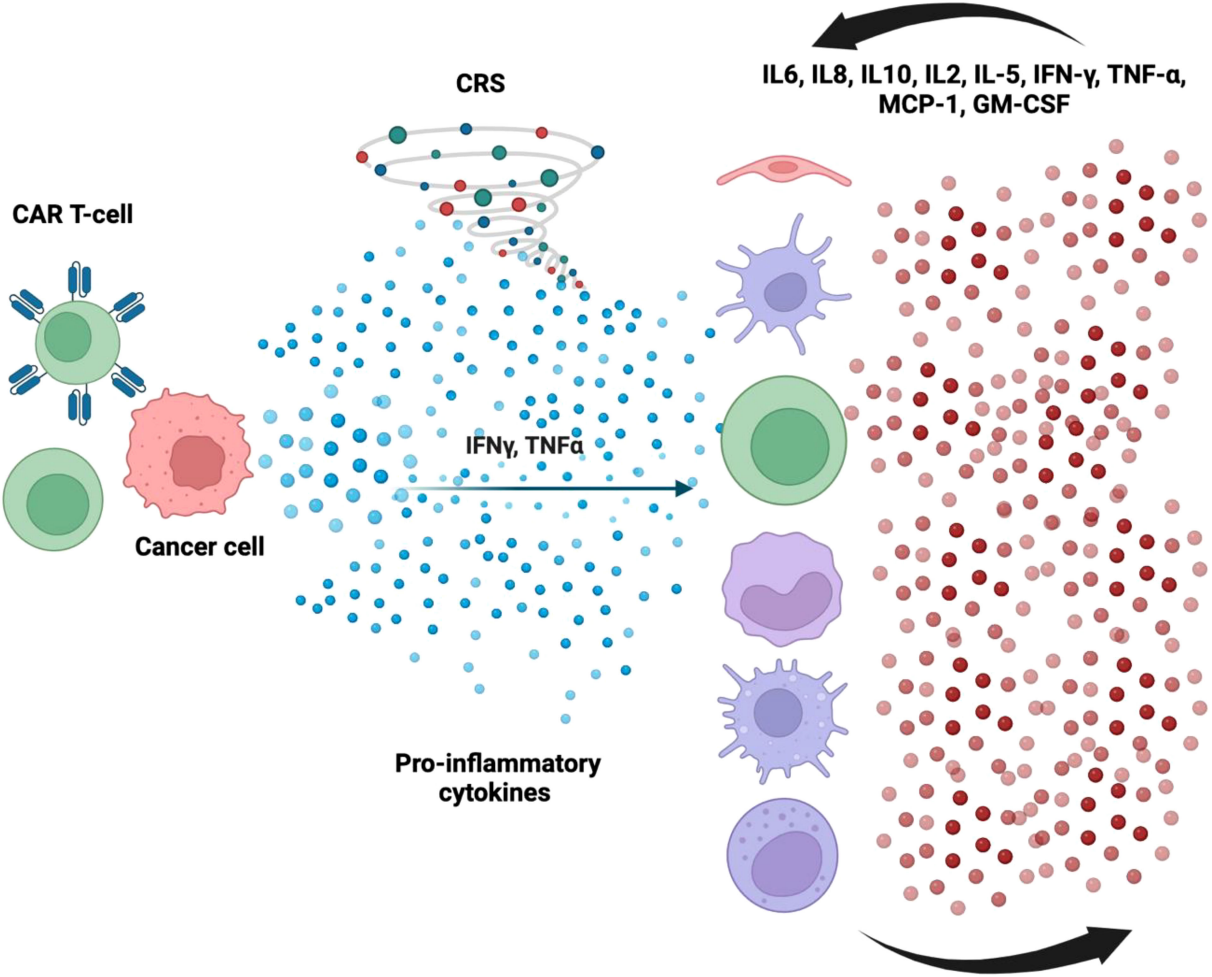
CAR T-cells target tumor cells and induce the release of cytokines as IFN-γ or TNF-α, which lead to the activation of bystander immune and non-immune cells as monocytes/macrophages, dendritic cells, NK and T-cell, and endothelial cells. These cells further release proinflammatory cytokines triggering a cascade reaction. Macrophages and endothelial cells produce large amounts of IL-6 which in turn activates T cells and other immune cells leading to a cytokine storm. Cytokine Release Syndrome (CRS) and myocarditis are interrelated inflammatory toxicities observed in patients undergoing Chimeric Antigen Receptor (CAR) T-cell therapy. Both conditions originate from immune system hyperactivation, leading to systemic and cardiac-specific inflammation (284). Upon administration, CAR T-cells recognize and bind to specific tumor antigens, triggering their activation and proliferation. This activation results in the release of pro-inflammatory cytokines such as interleukin-6 (IL-6), interleukin-1 (IL-1), interferon-gamma (IFN-γ), tumor necrosis factor-alpha (TNF-α), and granulocyte-macrophage colony-stimulating factor (GM-CSF). These cytokines further activate bystander immune cells, including monocytes and macrophages, amplifying the inflammatory response (285). IL-6 plays a pivotal role in the pathogenesis of CRS by promoting endothelial activation, increasing vascular permeability, and contributing to capillary leak syndrome. The resultant endothelial dysfunction can lead to hypotension, coagulopathy, and multiorgan failure. In the myocardium, this inflammatory milieu can cause direct cardiomyocyte injury, leading to myocarditis. Myocarditis in the context of CAR T-cell therapy is characterized by myocardial inflammation, which may result from direct cytotoxic effects of activated T-cells or from cytokine-mediated damage. Elevated levels of cytokines, particularly IL-6, TNF-α, and IFN-γ, have been associated with myocardial depression and arrhythmias. Additionally, endothelial activation within cardiac tissue can exacerbate myocardial injury through increased vascular permeability and infiltration of inflammatory cells (286). The interplay between systemic inflammation and cardiac-specific effects underscores the importance of early recognition and management of these toxicities. Therapeutic interventions targeting IL-6, such as tocilizumab, have shown efficacy in mitigating CRS symptoms and may also alleviate cardiac inflammation. However, further research is needed to delineate the precise mechanisms linking CRS and myocarditis and to develop targeted therapies that address both systemic and cardiac manifestations of CAR T-cell therapy-related toxicities. CAR, chimeric antigen receptor; IFN-γ, interferon-gamma; TNF-α, tumor necrosis factor-alpha; IL, interleukin; GM-CSF, granulocyte colony-stimulating factor; MCP-1, monocyte chemoattractant protein; NK cell, natural killer cell; DC, dendritic cell.

Even though CAR-T cell treatment has advanced significantly, its clinical use is often linked to major toxicities, including serious and sometimes fatal side effects, such as immunological effector cell-associated neurotoxicity syndrome (ICANS) and CRS (**Figure 2**) (37, 38). The adverse events of CRS types were shown in **Table 2**. Optimizing the clinical effectiveness of CAR-T cell therapy requires a thorough understanding of the unique features of each adverse event, as well as a methodical examination of how these events relate to the treatment. In this context, a great deal of research has been done to assess the negative consequences of CAR-T cell therapy, focusing mostly on CRS and neurotoxicity. Comparatively fewer studies have examined other possible side effects, such as cardiovascular toxicity, tumor lysis syndrome (TLS), and graft-versus-host disease (GVHD) (39–41). As a result, a comprehensive assessment of such side events is critical for mitigating or lowering their incidence in patients, permitting a more reasonable and tailored administration of CAR-T cell treatment in clinical practice. Cardiotoxicity refers to any direct or indirect detrimental effect on cardiac structure or function caused by a therapeutic agent. It may present as arrhythmias, myocardial infarction, heart failure, cardiomyopathy, pericardial disease, or changes in cardiac biomarkers or imaging parameters. Cardiotoxicity is often dose-dependent and may develop acutely, subacutely, or chronically. For example, decreased LVEF following CAR-T cell therapy or immune checkpoint inhibitors due to inflammation-mediated myocardial dysfunction (42, 43).

**Table 2:** The common terminology criteria for adverse events classifications for CRS.

Grade	Toxicity Description	Reference
Grade 1	Mild reaction, infusion interruption not indicated; intervention not indicated.	(57)
Grade 2	Therapy or infusion interruption indicated but responds promptly to symptomatic treatment (e.g., antihistamines, NSAIDs, narcotics, IV fluids); prophylactic medications indicated for ≤24 hours.	
Grade 3	Prolonged reaction (e.g., not rapidly responsive to symptomatic medication or brief interruption of infusion); recurrence of symptoms following initial improvement; hospitalization indicated for clinical sequelae (e.g., renal impairment, pulmonary infiltrates).	
Grade 4	Life-threatening consequences; pressor or ventilatory support indicated.	
Grade 5	Death.	

Among the various adverse effects linked to immunotherapy, cardiovascular toxicity has emerged as a particularly concerning yet underexplored issue. Earlier research has shown that immunotherapy-related cardiac complications pose a considerable risk of morbidity and mortality, which in turn hampers the progress and widespread clinical adoption of CAR-T cell therapy (44, 45). However, despite several studies exploring the cardiovascular toxicities associated with CAR-T cell therapy, the exact extent and characteristics of these toxic effects remain poorly understood (46). For example, 33 cardiovascular (CV) events, or 26% of the total, were discovered in a trial of 126 patients receiving CAR-T cell treatment that targeted antigens including CD19, CD22, and BCMA. Acute coronary syndrome (ACS), arrhythmias, and heart failure (HF) were the most often reported cardiovascular effects (47). Consequently, significant variations have often been observed in the total frequency and types of cardiovascular events that occur in cancer patients following CAR-T cell therapy (48–52).

**Figure 3:**
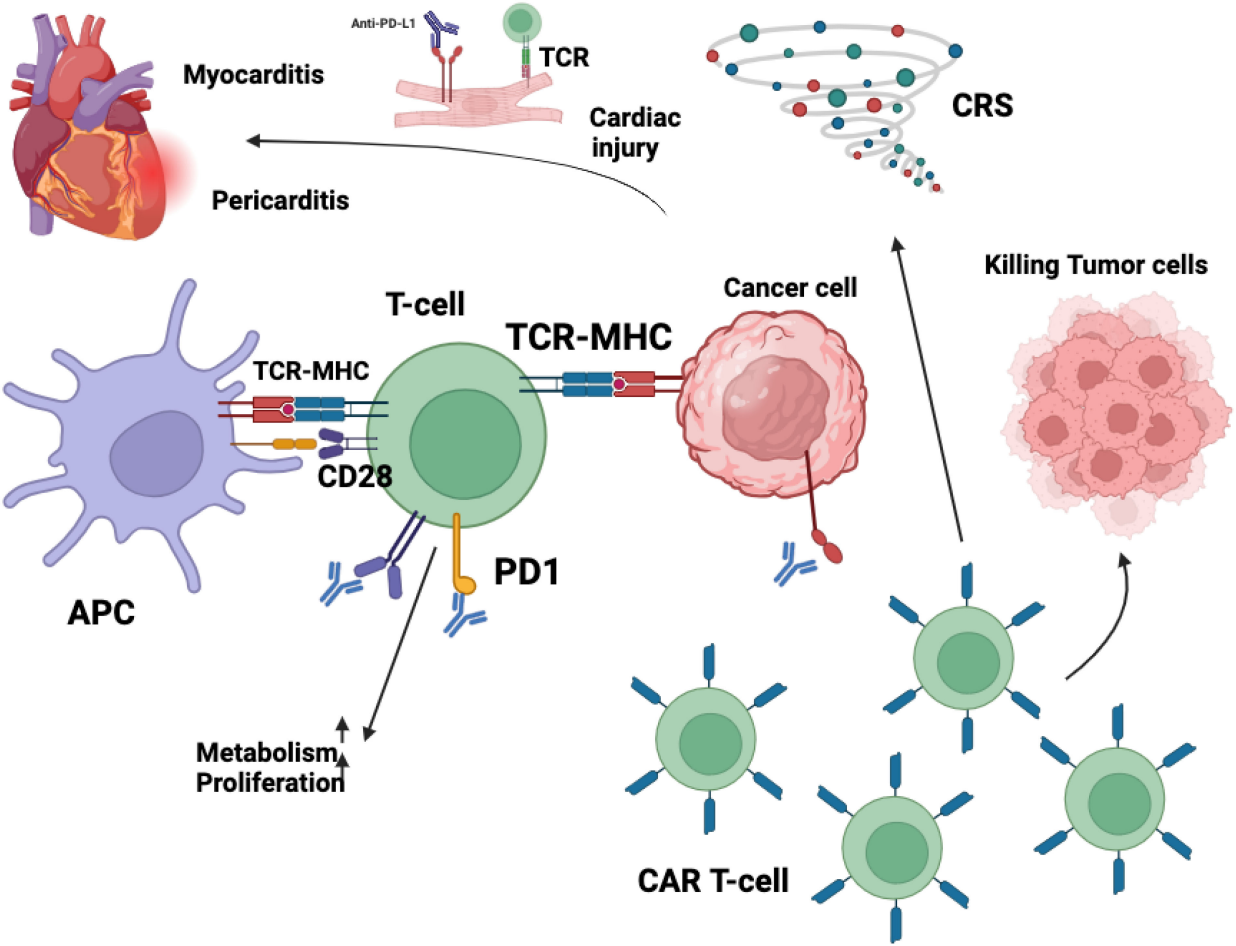
The cardiotoxic effects associated with immunotherapy arise from complex immune-mediated mechanisms that disrupt normal cardiac homeostasis. The blockade of cytotoxic T-lymphocyte-associated protein 4 (CTLA-4) signaling removes a critical checkpoint in immune regulation, leading to an unchecked expansion of cytotoxic CD8+ T lymphocytes. This dysregulated immune activation facilitates the infiltration of these effector cells into myocardial tissue, thereby contributing to localized inflammation and potential cardiomyocyte damage. Similarly, chimeric antigen receptor (CAR) T-cell therapy, while highly effective in targeting malignant cells, can precipitate significant immune-related adverse events. One of the most prominent complications is cytokine release syndrome (CRS), a hyperinflammatory response characterized by excessive production of proinflammatory cytokines. This systemic immune activation can exert profound effects on cardiovascular function, inducing endothelial dysfunction, increased vascular permeability, and myocardial inflammation, which may culminate in cardiotoxic manifestations such as myocarditis, arrhythmias, or even heart failure. Collectively, these immune-mediated processes underscore the necessity for close cardiac monitoring in patients receiving immunotherapy to mitigate the risk of severe cardiovascular complications. Immunotherapy, encompassing ICIs and CAR T-cell therapies, has significantly advanced cancer treatment but is associated with cardiovascular toxicities affecting the myocardium, vasculature, conduction system, and pericardium. ICIs function by inhibiting regulatory pathways such as PD-1/PD-L1 and CTLA-4, thereby enhancing T-cell-mediated anti-tumor responses. However, this immune activation can lead to unintended cardiac effects. Activated CD4^+^ and CD8^+^ T lymphocytes may infiltrate myocardial tissue, resulting in myocarditis characterized by inflammation and potential necrosis of cardiomyocytes (287). Additionally, ICIs may disrupt peripheral tolerance, leading to the production of autoantibodies against cardiac proteins like troponin I, contributing to autoimmune-mediated cardiac injury (288). The inhibition of PD-1/PD-L1 pathways, which are expressed on cardiomyocytes and endothelial cells, removes protective mechanisms against immune-mediated damage, further exacerbating cardiac inflammation. CAR T-cell therapies, designed to target specific tumor antigens, can also induce cardiovascular complications. These therapies may cause “on-target, off-tumor” effects if the targeted antigen is expressed on healthy cardiac tissues, leading to direct cardiotoxicity. Moreover, the activation of CAR T-cells can result in CRS, a systemic inflammatory response characterized by elevated levels of cytokines such as IL-6, TNF-α, and IFN-γ. These cytokines can have deleterious effects on the heart, including negative inotropy, endothelial dysfunction, and increased vascular permeability, culminating in hypotension, reduced myocardial contractility, and cardiomyopathy (258). In summary, while immunotherapies offer substantial benefits in oncology, their potential to cause significant cardiovascular adverse effects necessitates vigilant monitoring and management strategies to mitigate risks and ensure patient safety.

Two main processes are believed to be responsible for CAR-T cell toxicities: specific interactions between the CAR and its target antigen expressed by non-malignant cells, and a large systemic release of cytokines brought on by excessive T cell activation (53). CRS, a multisystem inflammatory reaction brought on by a cytokine storm from CAR-T cell infusion (**Figure 3**), is one of the most frequent side effects associated with CAR-T cell treatment. Among other toxic signs, 37-93% of lymphoma patients have CRS (54), and 77–93% of individuals diagnosed with leukemia (54–57). Clinical presentations of cytokine release syndrome can vary widely, from mild symptoms such as fever and general discomfort to more severe manifestations, including hypoxia, hypotension, organ damage, and, in extreme cases, the development of sepsis-like syndrome or fatal outcomes (55). According to theory, CRS results from the enormous and simultaneous activation of T cells, which causes a notable release of chemokines and proinflammatory cytokines (56, 58). Interleukins (IL)-6, IL-8, IL-10, and IL-15, as well as granulocyte-macrophage colony-stimulating factor (GM-CSF), interferon-gamma (IFN-γ), monocyte chemoattractant protein-1 (MCP-1), macrophage inflammatory protein-1 beta (MIP-1β), ferritin, and C-reactive protein (CRP), have all been linked to elevated levels of CRS. Elevated levels of soluble IL-2 receptor have also been noted in severe symptoms (54, 59). The management of CRS is tailored according to severity, with mild cases typically addressed through supportive interventions and the administration of antipyretics. In moderate CRS or cases refractory to initial supportive measures, therapeutic strategies include the use of interleukin-6 (IL-6) receptor antagonists such as tocilizumab. For more severe manifestations, corticosteroids, including dexamethasone, are employed to mitigate excessive immune activation and inflammatory responses (60, 61). In extreme cases, CRS may exhibit clinical signs similar to hemophagocytic lymphohistiocytosis (HLH) or macrophage activation syndrome (MAS). Anakinra, an interleukin-1 (IL-1) receptor antagonist, may be used for targeted immunomodulation as an alternate strategy to reduce hyperinflammatory reactions in situations where conventional therapeutic approaches are ineffective (62–65). Acute-phase reactants such as ferritin and C-reactive protein (CRP) are examples of serum inflammatory biomarkers that may be helpful clinical indicators for assessing treatment response or forecasting the onset of CRS. However, because cytokine levels are not readily available in clinical settings, real-time monitoring of these levels is still challenging (65).

ICANS, a disorder exhibiting a broad spectrum of clinical symptoms, has been connected with CRS. From mild cognitive impairment and confusion to severe neurological effects such as cerebral edema, seizures, and, in the worst situations, death, symptoms can vary widely (60, 66). Symptoms of CRS in the heart. Studies have shown a connection between increased levels of inflammatory cytokines like IL-6, IFN-γ, and TNFα and ICANS, even though the underlying mechanism of ICANS is not entirely understood in comparison to CRS (59, 67, 68). It is thought that these abnormal signaling pathways cause endothelial activation and dysfunction, which in turn causes the blood-brain barrier to be disrupted and vascular permeability to rise. Vigilant surveillance, regular neurological evaluations, and timely treatment intervention are necessary for effective management. Furthermore, sinus bradycardia, which is typically temporary and self-resolving and frequently doesn’t require any special therapy, has been linked to ICANS; yet, ongoing clinical monitoring is still crucial (69, 70). In addition, clinical observations have revealed a variety of systemic symptoms involving the constitutional, hematologic, renal, gastrointestinal, and dermatologic systems (67, 71–73).

Meta-analysis studies have demonstrated that cardiotoxicity is a frequently observed complication of CAR T-cell therapy, primarily driven by cytokine release syndrome. Therefore, rigorous monitoring and the implementation of individualized therapeutic strategies are essential to mitigate these adverse effects (74). A thorough investigation found that those receiving CAR-T cell treatment for cancer are more likely to experience cardiovascular side effects. The most common cardiovascular problems seen in these individuals were arrhythmias, circulatory system malfunction, and heart failure (75).

Recent meta-analyses have reported an overall incidence of cardiovascular events (CVEs) following CAR T-cell therapy ranging from 7% to 54% (74). However, these studies predominantly focus on patients with hematologic malignancies, such as those treated with CD19-targeted CAR T-cell therapies, where the incidence of CVEs is notably higher. A separate meta-analysis evaluating cardiovascular outcomes in CAR T-cell therapy recipients revealed that the occurrence of ventricular arrhythmias, myocardial infarction, and cardiovascular mortality was relatively uncommon during short- to mid-term follow-up periods. In contrast, left ventricular systolic dysfunction and supraventricular arrhythmias emerged as the most frequently reported cardiac events. These findings underscore the need for cardiovascular monitoring protocols that prioritize the early detection of declining ejection fraction and the surveillance of supraventricular arrhythmogenic activity in patients undergoing CAR T-cell therapy (76).

In contrast, data on CVEs in patients with solid tumors undergoing CAR T-cell therapy are limited, and available studies often report lower incidence rates. This discrepancy may be attributed to differences in tumor biology, patient selection, and the immunological milieu between hematologic and solid tumors (77). Furthermore, the heterogeneity in study designs, patient populations, and definitions of cardiovascular events contributes to variability in reported incidence rates. However, these aggregate rates do not fully capture the variability arising from differences in patient populations, particularly those with hematologic malignancies versus solid tumors.

#### 3.1.2 Preclinical evidence

Accumulating clinical evidence highlights that cardiovascular complications are a notable and potentially serious concern following CAR T-cell therapy. These events are frequently associated with CRS, an inflammatory cascade that is a common consequence of CAR-T infusion. Retrospective and prospective cohort studies across both pediatric and adult populations have reported a range of cardiovascular toxicities, including arrhythmias, heart failure, hypotension requiring inotropes, and cardiomyopathy, often in the context of CRS grade 2 or higher. Arrhythmias, particularly atrial fibrillation, are among the most frequently observed cardiac complications. Studies by Brammer et al. (78), Shouval et al. (79), and Lee et al. (80) documented that arrhythmia occurred in patients who predominantly experienced CRS, with many showing elevated levels of brain natriuretic peptide (BNP) and, to a lesser extent, troponin. In Lee et al.’s cohort, BNP elevation was significantly higher in patients with cardiac events, although troponin levels did not differ substantially between those with and without events. These findings suggest BNP may be a more sensitive marker for cardiac stress in this setting. Cardiomyopathy and reductions in left ventricular ejection fraction (LVEF) have also been consistently documented. Ganatra et al. (81) reported a median LVEF drop from 58% to 37% in patients who developed cardiomyopathy post-infusion. Similarly, Alvi et al. (82) and Korell et al. (83) observed declines in LVEF and noted that many of these cases were accompanied by elevated troponin and BNP levels. Notably, in Alvi’s cohort, all cardiovascular deaths occurred in patients with BNP levels exceeding 3,000 pg/mL, underscoring the prognostic significance of this biomarker. Furthermore, older patients and those with pre-existing cardiac risk factors were more likely to experience cardiac injury, although not all associations reached statistical significance. HF in both decompensated and new-onset was frequently linked to high-grade CRS. Studies by Lefebvre et al. (52), Alvi et al., and Ganatra et al. (81) each identified HF as a prominent complication, often occurring in tandem with arrhythmias or cardiomyopathy. Additionally, hypotension requiring vasopressor or inotropic support was commonly reported, especially in pediatric populations, as shown in studies by Burstein et al. (50) and Shalabi et al. In these cohorts, patients with baseline reduced LVEF or diastolic dysfunction had higher risks of developing hemodynamic instability, although age, sex, and race were not significant predictors. Importantly, CRS appears to be a central driver of these cardiac events. Multiple studies including those by Alvi, Ganatra, Lefebvre, and Korell demonstrated a strong correlation between CRS grade ≥2 and the incidence of cardiac complications. For example, in Alvi’s study, all 17 patients with cardiac events had CRS grade ≥2, and 83% of those with elevated troponin levels also had high-grade CRS. Moreover, CRS severity was associated with increased mortality, prolonged hospitalization, and the need for intensive care support in several cohorts. The concurrence of ICANS further elevated the risk of poor outcomes, suggesting a synergistic toxicity profile in severe systemic immune responses. While most cardiovascular events occurred early after CAR-T infusion, some delayed presentations were also noted. In Burstein et al., one pediatric patient experienced a cardiac arrest two months post-infusion. This underscores the need for continued cardiovascular surveillance, even after the acute CRS phase resolves. Although direct cardiovascular mortality was relatively rare, fatal outcomes were reported in Alvi et al. and Ganatra et al., particularly in patients with severe CRS, markedly elevated BNP, and pre-existing cardiac vulnerabilities. These deaths emphasize the importance of risk stratification, biomarker-guided monitoring, and prompt intervention.

### 3.2 ICIs

**Figure 4:**
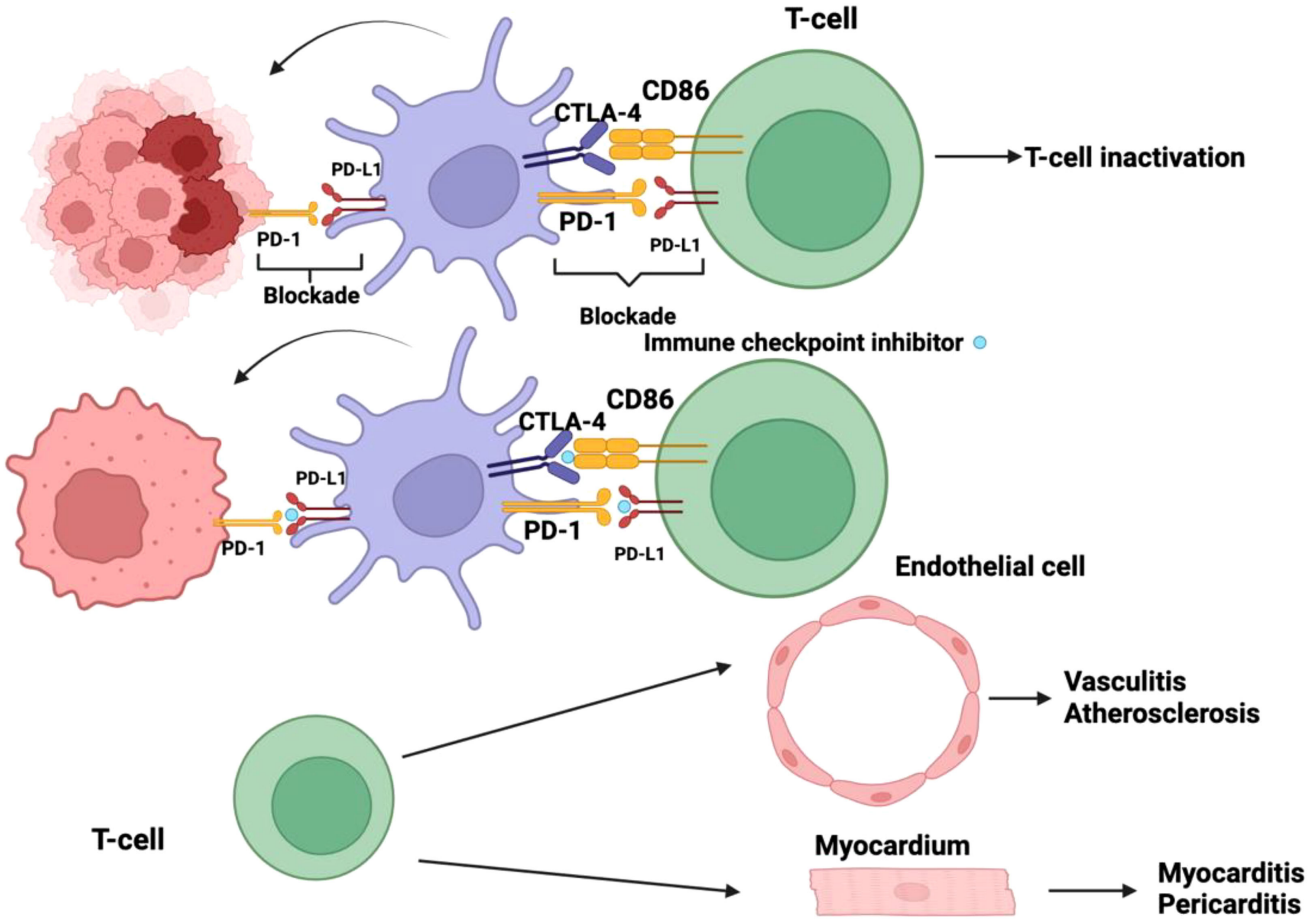
Checkpoint inhibitors play a pivotal role in immunotherapy by counteracting the immune evasion mechanisms employed by tumor cells. Malignant cells have evolved sophisticated strategies to suppress the host immune response, one of which involves exploiting immune checkpoint pathways. Specifically, the activation of programmed cell death protein-1 (PD-1) and cytotoxic T-lymphocyte-associated protein 4 (CTLA-4) receptors on dendritic cells (DCs) leads to their functional inactivation, thereby impairing antigen presentation and dampening T-cell priming. By blocking these inhibitory receptors, checkpoint inhibitors effectively prevent the immune-suppressive signaling cascade, allowing DCs to remain in an active state and sustain an anti-tumor immune response. Despite their therapeutic benefits, checkpoint inhibitors disrupt critical regulatory mechanisms that maintain immune homeostasis. The inhibition of immune checkpoints diminishes self-tolerance, increasing the risk of immune-mediated tissue damage. This loss of immunological regulation may lead to autoreactive T-cell activation, resulting in off-target effects where the immune system indiscriminately attacks host tissues. Notably, the unintended targeting of endothelial cells can precipitate inflammatory vascular conditions such as vasculitis and may contribute to the development or exacerbation of atherosclerosis. Furthermore, immune-related adverse effects can extend to cardiac tissues, where T-cell-mediated aggression against myocardial or pericardial cells may manifest as myocarditis or pericarditis. These immune-related toxicities underscore the need for vigilant monitoring and appropriate management strategies to balance the therapeutic efficacy of checkpoint blockade with the potential risks of autoimmunity.

In 1996, Leach et al. announced the first discovery of a ‘immune checkpoint’ molecule, cytotoxic T-lymphocyte-associated antigen 4 (CTLA-4), which was a significant breakthrough in immunological research (84, 85). In a ground-breaking work, they showed that in a syngeneic mouse system, the injection of a CTLA-4-targeting antibody in a murine model successfully increased tumor rejection (85). This pivotal finding enabled a first-in-human Phase I clinical trial evaluating CTLA-4 inhibition (MDX-010) in patients with ovarian and melanoma cancer, and the results were encouraging. Later Phase II investigations in melanoma proceeded fast, resulting in a landmark Phase III study that served as the foundation for the FDA’s approval of ipilimumab (**Table 3**), a humanized anti-CTLA-4 monoclonal antibody, for the treatment of metastatic melanoma (86–88). These trials yielded a number of significant findings, confirming immune checkpoint blockage as a distinct and cutting-edge cancer therapy method (**Figure 4**). Notably, immunotherapy was not directly comparable to the typical response kinetics used to assess anti-cancer medicines, which mostly rely on the assessment of tumor shrinkage (84). This family of therapeutic drugs has a distinct immune-related response pattern that includes an initial increase in tumor size, followed by eventual shrinkage and, in some cases, the brief emergence of new lesions. As a result, a unique response criterion tailored to immunotherapy was developed. Furthermore, the ‘tail of the curve’ refers to the tendency of patients who improve following medication to maintain a protracted response, which typically lasts for years (84). This is believed to be an example of immunologic memory formation in the context of cancer immunotherapy. Ipilimumab was authorized ten years ago to treat metastatic melanoma (89). Dr. Allison was awarded the Nobel Prize for his groundbreaking discovery of CTLA-4, and the development of ipilimumab has revolutionized melanoma treatment. Nowadays, it is frequently used in combination with nivolumab (an anti-PD-1 drug), which gives metastatic melanoma patients a five-year survival rate of over 50%. In the age of cancer immunotherapy, this finding calls into question what a “cure” actually means.

**Table 3:** FDA-approved ICIs and their targets.

ICI Name	Target	Approved Cancers	Reference
Ipilimumab	CTLA-4	Melanoma, Colorectal Cancer (CRC), HCC, NSCLC, RCC	(44, 252–256)
Nivolumab	PD-1	Melanoma, NSCLC, Renal cell carcinoma (RCC), Hodgkin’s lymphoma	
Pembrolizumab	PD-1	Melanoma, NSCLC, Head & Neck SCC	
Atezolizumab	PD-L1	NSCLC, Urothelial Carcinoma	
Durvalumab	PD-L1	NSCLC, Small Cell Lung Cancer	
Relatlimab	LAG-3	Advanced solid tumors	

The second immune checkpoint molecule discovered was anti-PD-1, whose primary ligand, PD-L1, was first identified by Dr. Tasuko Honjo and his colleagues in 2000. He received the Nobel Prize as a result of this discovery (90). This finding was further reinforced by mouse studies conducted by Iwai, Honjo, and their colleagues, which demonstrated that tumor formation was inhibited in animals lacking anti-PD-1. Furthermore, similar outcomes were observed in immunocompetent wild-type mice given anti-PD-L1 antibodies (91, 92). Phase I clinical trials were initiated when anti-PD-1 and anti-PD-L1 monoclonal antibodies showed therapeutic effectiveness against a range of tumor types, including renal cell carcinoma, melanoma, and non-small-cell lung cancer (NSCLC) (93). Single-agent anti-PD-(L)1 therapies have been authorized and are currently being used extensively for over 15 cancer indications, including solid tumors and hematologic malignancies, as a result of the quick advancement of clinical trials in these and other tumor types (94). Combining anti-PD-1 and CTLA-4 inhibition, primarily by partnering ipilimumab with nivolumab, has been approved for several of indications, including advanced non-small cell lung cancer, melanoma, and kidney cancer. This combination is also being researched as an adjuvant and neoadjuvant treatment for illnesses that are still in the early stages of sickness. Anti-PD-1 treatments that are tumor-agnostic have been authorized based on the molecular characteristics of tumors, such as those with a high burden of non-synonymous mutations, as shown by microsatellite instability-high colorectal and endometrial malignancies (95). These findings support the hypothesis that more complex tumors can trigger a more varied immune response, hence enhancing anti-tumor immunity. Immune-related adverse events (irAEs) are defined as adverse effects arising from activation or dysregulation of the immune system, particularly in the context of ICI therapy. These may involve cardiac tissues (e.g., ICI-induced myocarditis) or other organs such as the lungs, liver, or skin.

It was anticipated that utilizing the immune system in this manner would result in a variety of side effects, which are often known as irAEs, as ICIs disrupt immunological regulatory systems (**Table 4**) (96). 70–90% of individuals have these adverse events, and all organs may be affected (96). Clinical symptoms often manifest in the initial weeks to months following the initiation of treatment, and 10 to 15 percent of patients develop severe immune-related adverse effects. However, these symptoms might show up years after therapy is over or at any time (97). The incidence and intensity of irAEs are increased by combination immunotherapy, such as the anti-PD-1/PD-L1 and CTLA-4 regimen. Little is known about other risk factors for irAEs since high-risk individuals are excluded from comprehensive clinical research. As a result, it is still uncertain how safe ICIs are for people with autoimmune diseases, solid organ recipients, and hematopoietic stem-cell transplant recipients.

**Table 4:** Immune-related cardiovascular adverse events (irAEs) that variety study reported.

Adverse Event	Incidence Rate	ICIs Involved	Reference
Myocarditis	0.04% - 1.14%	Anti-PD-1, Anti-CTLA-4	(152, 172)
Heart Failure	5.6% - 6.1%	Anti-PD-1, Anti-CTLA-4, Anti-PD-L1	(155, 257)
Pericarditis	10.94% (7/64)	Anti-PD-1, Anti-PD-L1	(149)
Myocardial infarction	17.19% (11/64)	Anti-PD-1, Anti-PD-L1	(149)
Arrhythmias	53.13% (34/64)	Anti-CTLA-4, Anti-PD-1	(149)

#### 3.2.1 CTLA-4 mechanism

CTLA-4, also known as CD152, was one of the first negative regulatory molecules to be identified and targeted in therapeutic situations (98). The majority of CTLA-4 is found on Foxp3+ regulatory T lymphocytes (Tregs), which mainly control the degree of early T cell activation, and on normal T cells throughout activation (99–102). Ipilimumab (anti-CTLA-4 monoclonal antibody) is currently used to treat a variety of cancers, such as melanoma, colorectal cancer (CRC), hepatocellular carcinoma (HCC), non-small cell lung cancer (NSCLC), and renal cell carcinoma (RCC), either by itself or in combination with nivolumab (anti-PD-1 monoclonal antibody) (103).

The way CTLA-4 works is by blocking CD28, a T cell co-stimulatory receptor (104–106), and shares ligands [CD80 (B7.1) and CD86 (B7.2)] with CD28 (107–111). One theory is that CTLA-4’s greater affinity for CD80 and CD86 lowers T cell activation by displacing CD28 from these receptors, even if the precise mechanisms behind its impact remain unknown (112–118). Immunological suppression results from CTLA-4’s activation of inhibitory signaling through the protein phosphatases SHP2 and PP2A when it binds to CD80 and CD86 on antigen-presenting cells (119), including general immunosuppression, reduced cytokine release, increased Treg activity, decreased T lymphocyte proliferation, and inhibition of adaptive responses (120–122). Furthermore, it has been shown that ipilimumab (anti-CTLA-4 mAb) uses antibody-dependent cell-mediated (ADCC) cytotoxicity to selectively deplete CTLA-4+ FOXP3+ Treg cells (99).

#### 3.2.2 LAG-3 mechanism

This molecule was identified as a homologue of CD4 more than 20 years ago, and it has since attracted a lot of interest (123). When LAG-3 was shown to enhance Treg cell activity in 2005, its function as an immune checkpoint became clear (124, 125). In addition to its action on Treg cells, LAG-3 itself suppresses CD8+ effector T cells (126). The only known ligand for LAG-3 is MHC class II molecules, which are elevated on some epithelial cancers in response to IFN-γ. These substances are also seen on tumor-infiltrating dendritic cells and macrophages (112). LAG-3 antibodies that do not interfere with the LAG-3-MHC class II interaction have been demonstrated to enhance T cell proliferation and promote effector cell functions both *in vitro* and *in vivo*. However, the precise role of this interaction in suppressing T cell responses remains incompletely understood (112). It is still largely unknown what the molecular mechanisms govern LAG-3 signaling and how it interacts with other immunological checkpoints (127). In a range of biological contexts, LAG-3 and PD-1 have demonstrated a potent functional synergy that inhibits immune responses (128, 129). The combination of relatlimab, a monoclonal antibody targeting LAG-3 (BMS-986016), and nivolumab, a monoclonal antibody against PD-1, has demonstrated significant therapeutic efficacy in melanoma patients who have previously shown resistance to anti-PD-1/PD-L1 treatments (130). A significant upregulation of LAG-3 expression was observed in several malignancies when compared to their corresponding normal tissue counterparts. This includes kidney renal clear cell carcinoma (KIRC), pancreatic adenocarcinoma (PAAD), skin cutaneous melanoma (SKCM), testicular germ cell tumors (TGCT), lymphoid neoplasm diffuse large B-cell lymphoma (DLBC), and head and neck squamous cell carcinoma (HNSC). The elevated expression of LAG-3 in these cancers suggests its potential role in the immune evasion mechanisms of tumors, which may contribute to their progression and resistance to immune responses. This finding underscores the importance of LAG-3 as a promising target for immunotherapeutic interventions in these malignancies (131). This suggests that LAG-3 targeting may have a strong antitumor effect in some cancer types (131).

#### 3.2.3 PD-1/PD-L1

PD-1 and PD-L1 inhibitors enable the prolonged activation of T lymphocytes, improving their capacity to eradicate tumor cells by inhibiting inhibitory signaling pathways (91, 132). PD-1 is present on a variety of immune cells, such as monocytes, T cells, B cells, dendritic cells (DCs), and tumor-infiltrating lymphocytes (TILs) (133, 134). However, PD-L1 is usually present on tumor cells and APCs (134). PD-1 has been demonstrated to be expressed by a wide range of human cancers, including ovarian neoplasms, bladder carcinoma, head and neck squamous cell carcinoma, lung carcinoma, renal cell carcinoma (RCC), melanoma, and gastrointestinal cancers (135). PD-1 is essential for controlling T cell activity in peripheral tissues, especially during inflammatory reactions, and it also helps control autoimmune diseases. CTLA-4, on the other hand, mostly controls T-cell proliferation in lymphoid organs during the early stages of immunological activation (136–142). The difference between CTLA-4 and PD-1/PD-L1 inhibitors is shown in **Table 5**.

**Table 5:** Comparative overview of cardiovascular toxicities: CTLA-4 vs. PD-1/PD-L1 Inhibitors.

Feature	CTLA-4 Inhibitors	PD-1/PD-L1 Inhibitors	Reference
Common Agents	Ipilimumab	Nivolumab, Pembrolizumab, Atezolizumab	(258–267)
Primary Cardiovascular Toxicity	Myocarditis	Atherosclerosis progression	
Incidence of Myocarditis	Approximately 0.27%–1.14%; higher with combination therapy	Similar incidence; increased risk with combination therapy	
Mortality Rate of Myocarditis	Up to 50%	Comparable mortality rates	
Mechanisms	- T-cell and macrophage infiltration into myocardium- Elevated pro-inflammatory cytokines (e.g., IFN-γ, TNF-α, IL-17)- Loss of regulatory T-cell function- Endothelial activation via ICAM-1 expression	- T-cell-mediated vascular inflammation- Increased IL-17 signaling- Shift from macrophage to lymphocyte-dominated plaque inflammation- PD-1 deficiency leading to elevated cholesterol synthesis genes	
Clinical Manifestations	- Arrhythmias- Heart failure- Pericarditis- Vasculitis	- Accelerated atherosclerosis- Acute coronary syndrome- Myocardial infarction- Hypertension	
Time to Onset	Median of 17–34 days post-initiation	Variable; atherosclerotic changes may develop over months	
Response to Immunosuppression	Often resistant; may require agents like abatacept	Variable; management strategies are still being defined	
Combination Therapy Risk	Increased risk and severity of myocarditis with CTLA-4 and PD-1 inhibitors combined	Combination therapy associated with higher incidence of cardiovascular events	

A vital immunosuppressive mechanism in the tumor microenvironment (TME), PD-1 promotes immune evasion and increases tumor resistance to immune surveillance (143–145). By blocking the stimulatory signaling cascades triggered by the interaction between TCR and CD28, PD-1 expression inhibits the activation of critical transcription factors required for T cell activation, proliferation, effector function, and survival (98). T-cell viability is ultimately compromised by this regulatory mechanism, which includes the suppression of anti-apoptotic molecules like Bcl-2 and Bcl-xL as well as the inhibition of important transcription factors like activator protein 1 (AP-1), nuclear factor of activated T cells (NFAT), and nuclear factor-κB (NF-κB) (146). A negative regulatory feedback system is started by the activation of PD-1 ligands, which attenuates the production of cytokines and suppresses immunological responses (119). Like CTLA-4, PD-1 is abundantly expressed on Tregs, which may increase their ability to proliferate when it interacts with its matching ligand (147). A meta-analysis of 20 clinical trials demonstrated that CTLA-4 inhibitors significantly increased the risk of all-grade cardiovascular toxicities (OR = 1.33, 95% CI: 1.00–1.75, p = 0.05), with an even higher risk observed for patients receiving monotherapy (OR = 1.73, 95% CI: 1.13–2.65, p = 0.01). Furthermore, CTLA-4 inhibitors were associated with a twofold increase in the incidence of grade 3–5 cardiovascular adverse events (OR = 2.00, 95% CI: 1.08–3.70, p = 0.03). Although higher rates of heart failure, hypertension, pericardial effusion, myocarditis, and atrial fibrillation were observed, these did not reach statistical significance when analyzed individually (148).

#### 3.2.4 Cardiovascular and immune checkpoint complications

Mortality rates in severe instances vary from 10% to 17%, however, most irAEs may be efficiently controlled in their early stages (149, 150). The accompanying fatality rate is shockingly high, even though the frequency of cardiovascular adverse events caused by ICIs is quite low (151). Heart irAEs gained widespread attention after Johnson et al. (153) reported two incidences of deadly myocarditis after ICI treatment (152, 153). Over time, a wide range of clinical conditions have been recognized as immune-related adverse events (irAEs) impacting the cardiovascular system. These include acute myocardial infarction, conduction disturbances like atrioventricular (AV) block, ventricular and supraventricular arrhythmias, sudden cardiac arrest, and Tako-Tsubo cardiomyopathy. Other cardiovascular complications include non-inflammatory cardiomyopathy, pericardial disorders such as pericarditis and pericardial effusion, cerebrovascular issues like ischemic stroke, thromboembolic events such as venous thromboembolism, and the accelerated progression of atherosclerosis (154). A retrospective study of 424 individuals who received at least one ICI found that 62 patients (14.6%) experienced newly identified cardiovascular issues after beginning ICI treatment (155). In this cohort, 5.6% of patients experienced heart failure while undergoing treatment with ICI monotherapy (**Table 6**), highlighting the potential cardiovascular risks associated with this therapeutic approach. The occurrence of heart failure in this patient group underscores the need for careful monitoring and management of cardiovascular health during ICI-based treatments (155). When two ICIs were given consecutively, the incidence of heart failure increased to 6.1% (155). A similar pattern was seen in a recent meta-analysis that comprised 13,646 patients receiving anti-CTLA-4, anti-PD-1, and/or anti-PD-L1 treatments (156). The subgroup of patients receiving ICI therapy as the only treatment method had a 3.1% incidence of cardiovascular adverse events (156). It is significant to note that the incidence of cardiovascular adverse events in patients undergoing combination immunotherapy almost doubled, reaching 5.8% (156). When chemotherapy and ICI treatment were introduced, the incidence rate was constant at 3.7% with no discernible change (156). These findings are consistent with evidence from clinical trials, which indicate that CTLA-4 inhibition is particularly associated with an increased risk of immune-mediated cardiotoxicity, which encompasses conditions such as pericarditis and myocarditis (157, 158). Similarly, cases of myocarditis have been documented when anti-PD-1 medications like nivolumab or pembrolizumab have been administered (159, 160). The relationship between the use of ICIs and an increased risk of cardiovascular issues, including myocarditis, pericardial diseases, heart failure, dyslipidemia, myocardial infarction, and cerebral arterial ischemia, was further substantiated by an independent meta-analysis conducted by Dolladille et al. This study, which analyzed data from 32,518 patients, reinforced the evidence that ICI treatment is associated with a higher incidence of these serious cardiovascular conditions, highlighting the need for vigilant monitoring and early intervention in patients undergoing ICI therapy (161). The “number needed to harm” (NNH), which measures how many people must be exposed to a specific treatment or risk factor for one person to experience an adverse event, was 462 for myocarditis and 260 for heart failure. It’s interesting to note that heart failure was one of the more frequent adverse events among patients receiving ICIs (161).

**Table 6:** Comparison of monotherapy vs. combination therapy in cardiovascular risk.

Therapy Type	Cardiac irAE Risk	Mortality Rate	Reference
Anti-PD-1 Monotherapy	Low	36%	(162)
Anti-CTLA-4 Monotherapy	Moderate	27%	(268)
Anti-PD-1 + Anti-CTLA-4 Combination	High	67%	(162)

In recent years, there has been a growing awareness of the link between ICIs and myocarditis, an inflammation of the heart muscle. While ICIs have proven effective in treating various cancers, this connection highlights the potential risk of immune-related damage to the heart. Increased recognition of this complication emphasizes the importance of careful cardiac monitoring and early detection in patients receiving ICI therapy, helping to reduce the severity of myocarditis and enhance patient outcomes (162–164). Numerous preclinical investigations have shown a connection between ICI treatment and myocarditis (165–170). The fact that proactive checks for myocarditis were not a part of the early research on ICIs raises worries that some instances may have gone unnoticed (171). Myocarditis is said to be associated with ICI treatments in between 0.04% and 1.14% of cases (152, 172). The most often reported cardiac adverse event is myocarditis, mostly because of its substantial impact on morbidity rates (173–175). Compared to other irAEs, myocarditis has a much higher mortality rate, ranging from 25% to 50% (153, 162, 172, 174).

The use of combination ICI therapy is a risk factor for myocarditis linked to ICIs (152). For example, using ipilimumab (anti-CTLA-4 monoclonal antibody) and nivolumab (anti-PD-1 monoclonal antibody) together increases the risk of myocarditis by 4.74 times when compared to using nivolumab just by itself (153). Recent clinical trials examining the combination of relatlimab (an anti-LAG-3 monoclonal antibody) and nivolumab (an anti-PD-1 monoclonal antibody) have shown a slight increase in the incidence of myocarditis. Specifically, myocarditis occurred in 1.7% of patients receiving the combination therapy, compared to 0.6% in those treated with nivolumab alone. This modest rise suggests that combining these immune checkpoint inhibitors may elevate the risk of immune-related cardiac inflammation. These results highlight the importance of vigilant cardiac monitoring in patients undergoing combination ICI treatment, as this adverse event, while uncommon, could have serious clinical consequences. Further research is needed to understand the mechanisms driving this increased risk and to improve patient care (176). Moreover, myocarditis resulting from combination therapies has been associated with higher mortality rates and increased severity when compared to cases induced by monotherapy. This suggests that the simultaneous targeting of multiple immune checkpoint pathways may enhance the intensity of the immune response, potentially leading to more aggressive forms of myocarditis. The heightened risk of severe outcomes underscores the importance of early detection and proactive management in patients undergoing combination immunotherapy, as the potential for life-threatening complications is more pronounced (162). Research found that the combination of anti-PD-1/PD-L1 and anti-CTLA-4 medicines resulted in a considerably higher fatality rate of 67%, compared to the 36% mortality rate seen with anti-PD-1/PD-L1 monotherapy (162). However, considering that the study only included 59 individuals, it is crucial to recognize that the observed fatality rate could not fully represent the actual risk.

According to studies, mice with defective thymic selection may develop autoreactive T cells specific to cardiac myosin in a naïve condition. These cells have higher expression levels of PD-1 to reduce the risk of autoimmune responses (170, 177). In our mouse model, autoreactive T lymphocytes that target cardiac tissue appear to be directly activated when the inhibitory PD-1/PD-L1 pathway is blocked by anti-PD-1 monoclonal antibody therapy. IFN-γ, perforin, and granzyme B are among the cytotoxic molecules and effector cytokines that are overproduced as a result of this activation, which eventually causes cardiac damage and the development of myocarditis (170). Patients with ICI-induced myocarditis showed clonal growth of T lymphocytes specific to cardiac myosin in their peripheral blood mononuclear cells, indicating their harmful role in the condition’s clinical presentation (178, 179).

While the link between anti-PD-1 monoclonal antibody therapy and ICI-related myocarditis is well established, the involvement of anti-CTLA-4 monoclonal antibody treatment in causing this condition remains uncertain. It has yet to be definitively proven whether anti-CTLA-4 therapy triggers autoreactive T cells that attack cardiac myosin heavy chain. Nonetheless, preclinical studies offer valuable insights: for example, CTLA-4-deficient mice develop fatal myocarditis early in life, underscoring the essential role of CTLA-4 in maintaining immune tolerance to cardiac tissue (180, 181). The loss of CTLA-4 function appears to facilitate the activation of autoreactive T cells, which could lead to immune-mediated damage in the heart. This suggests that inhibition of CTLA-4 may disrupt immune regulation, promoting the emergence of pathogenic T cells that target cardiac components. These findings underscore the complexity of immune responses induced by ICI therapy and the need for further investigation into the molecular mechanisms underlying CTLA-4-mediated immune tolerance in the context of autoimmune myocarditis (151). Furthermore, cardiac myosin-specific T cells showed higher levels of CTLA-4 expression than bystander T cells in our animal model of ICI-induced myocarditis (170, 182). Therefore, by activating autoreactive T cells that target cardiac myosin in clinical settings, blocking CTLA-4 with monoclonal antibodies may cause severe myocarditis, much like PD-1 inhibition.

Several studies in preclinical and clinical models have demonstrated that Th17 cells, or T cells that produce IL-17, are essential to the pathogenesis of various forms of myocarditis (183–186). These cells have a role in the development of dilated cardiomyopathy after acute myocarditis and are significant inflammatory mediators (183–185, 187). Importantly, it has been demonstrated that severing the connection between CTLA-4 and B7 molecules stimulates the growth of Th17 cells both *in vitro* and *in vivo*, as well as Th17-mediated autoimmunity in mice models (186). Furthermore, a number of studies have emphasized CTLA-4’s function in preserving peripheral heart tolerance. All of these results point to the possibility that blocking CTLA-4 pathways might precipitate myocarditis and exacerbate its severity.

Due to LAG-3’s recent FDA approval, there are a few cases of ICI-induced myocarditis linked to it. Previous LAG-3-related animal research has demonstrated that mice lacking LAG-3 did not develop myocarditis (188). However, when both LAG-3 and PD-1 were lacking, a severe type of myocarditis developed that was marked by T-cell infiltration, increased production of tumor necrosis factor (TNF), and persistent Treg inhibitory activity (189). Although considerable progress has been made in elucidating the pathophysiological mechanisms underlying certain ICI-related cardiovascular toxicities, particularly myocarditis and atherosclerosis, our understanding remains limited for other manifestations such as arrhythmias, heart failure, vasculitis, pericardial disorders, and venous thromboembolism. Nonetheless, emerging data suggest that a set of shared immunological pathways may contribute to the broader spectrum of cardiovascular adverse events induced by ICIs. Central to these processes is the enhanced activation of T lymphocytes following checkpoint inhibition, which results in elevated secretion of pro-inflammatory cytokines, including IL-1β, IL-6, and IL-17, as well as IFN-γ and TNF-α. These cytokines are increasingly recognized as key mediators of inflammatory damage to cardiovascular tissues. Additionally, crosstalk between activated T cells and macrophages appears to play a crucial role in amplifying inflammatory signaling and sustaining immune-mediated injury. Recent clinical and experimental findings have also implicated the thymus as a potential central modulator of ICI-associated cardiotoxicity. Specifically, structural or functional abnormalities of the thymus have been observed in patients with myocarditis, and animal studies have demonstrated that perturbations in thymic cytokine signaling can be associated with cardiac dysfunction. A deeper understanding of these converging mechanisms may inform the development of targeted cardioprotective interventions. However, the application of such strategies must be carefully weighed against the potential risk of dampening the intended anti-tumor immune response, which is essential for the therapeutic efficacy of ICIs (190).

### 3.3 Vaccine

#### 3.3.1 mRNA vaccine

Recent studies examining the safety profile of mRNA vaccines for COVID-19 have drawn attention to the occurrence of cardiovascular complications. However, there remains a notable paucity of research exploring the potential cardiovascular effects of mRNA vaccines in cancer immunotherapy. A comprehensive analysis conducted using the World Health Organization’s (WHO) global pharmacovigilance database, VigiBase, found that recipients of the BNT162b2 (Pfizer-BioNTech) COVID-19 vaccine exhibited the highest reported incidence of cardiovascular adverse events. Notably, the study found that the BNT162b2 and mRNA-1273 (Moderna) vaccines were associated with 30% and 44%, respectively, of the reported cardiovascular-related adverse events following vaccination. The most frequently reported cardiovascular symptoms for both vaccines were palpitations and tachycardia, which are indicative of altered heart rhythm and could suggest an underlying cardiovascular stress response. This data highlights the critical need for vigilant cardiovascular monitoring in individuals receiving mRNA-based vaccines, especially as these technologies are increasingly applied in cancer immunotherapy. Considering the distinct mechanisms by which mRNA vaccines operate, further research is essential to better understand their long-term effects and potential risks, particularly in patients with existing cardiovascular conditions (191). Furthermore, Simone et al. documented a very uncommon case of myocarditis (n = 15) in a sizable cohort of people who had never had heart disease before after receiving the mRNA COVID-19 vaccine (192). Likewise, another extensive medical investigation found a connection between the duration of myocarditis in young males and the period after receiving the Pfizer-BioNTech vaccine (193).

Although the precise pathophysiology of CV symptoms following COVID-19 vaccination remains unclear, some studies propose potential mechanisms for these adverse events. It is suggested that mRNA vaccination may trigger an immune response in genetically predisposed individuals, potentially leading to the recognition of mRNA as an antigen. This immunomodulatory reaction may result in myocarditis and other systemic effects, driven by the activation of inflammatory pathways after dendritic cell and Toll-like receptor stimulation, which induces cytokine production (194, 195). Similarly, in those with moderate, “compensated” thrombocytopenia or chronic, hereditary thrombocytopenia, increased macrophage activity and reduced platelet production may be the cause of post-vaccination immune thrombocytopenic purpura (ITP) (196, 197). The development of thrombosis in unusual locations, such as the splanchnic, adrenal, cerebral, and ophthalmic veins, is a characteristic of immune thrombocytopenia (ITP) after vaccination, according to postmortem examinations of individuals with vaccine-induced thrombotic thrombocytopenia (VITT) (198).

The Israeli Ministry of Health revealed in late April 2021 that myocarditis instances had been reported among recipients of the BNT162b2 (Pfizer-BioNTech) vaccination (199). The FDA has documented 45 reported cases of myocarditis in its Vaccine Adverse Event Reporting System (VAERS), with 19 of these cases linked to the Pfizer-BioNTech vaccine and 26 attributed to the Moderna vaccine. In response to these reports, the Centers for Disease Control and Prevention (CDC) initiated a comprehensive investigation aimed at assessing the long-term effects of myocarditis following vaccination with the two authorized mRNA COVID-19 vaccines. The study involved surveying individuals who had experienced myocarditis post-vaccination to better understand the lasting impact of these cardiac events on overall health. Alongside myocarditis, the mRNA vaccines have been associated with a range of other serious cardiovascular complications, including thrombosis, thrombocytopenia, and stroke. These findings underscore the importance of continuous monitoring and vigilance for cardiovascular side effects in individuals receiving mRNA-based vaccines. As the application of mRNA vaccines extends into emerging fields like cancer therapy, it becomes increasingly vital to assess the broader implications. Careful evaluation of the benefits versus potential risks is especially important for those with pre-existing cardiovascular conditions (199). Many of the cardiovascular effects reported following mRNA vaccination, such as myocarditis, are based on case reports and data from post-marketing surveillance systems like VAERS or national health databases. While these systems play a vital role in identifying potential safety signals, they are subject to underreporting, reporting biases, and a lack of comprehensive clinical detail. Therefore, findings from such data should be interpreted with caution, as they may not be fully generalizable. Confirmatory evidence from prospective, controlled studies is essential to accurately characterize the incidence, risk factors, and outcomes associated with these events. In addition, an association of mRNA vaccine and myocarditis, such as long-term follow-up results, and gender/age-specific risks is shown in **Table 7**.

**Table 7:** Incidence and outcomes of myocarditis after BNT162b2 vaccination (mRNA vaccine).

Parameter	Findings	Reference
Highest Risk Group	Males aged 16–19 years	(200)
Incidence in Males 16–19 Years	150.7 cases per million	(269)
Incidence in Females 16–19 Years	10 cases per million	(269)
Incidence After Second Dose in Males 16–17 Years	15.7 cases per 100,000 doses	(270)
Median Time to Symptom Onset	2–3 days post-vaccination	(271)
Severity	Majority mild; <1% severe cases	(200)
Long-Term Outcomes	Lower frequency of cardiovascular complications compared to conventional myocarditis at 18 months	(272)

Myocarditis has been identified as a rare but notable adverse event following administration of the BNT162b2 (Pfizer–BioNTech) mRNA COVID-19 vaccine, with incidence and severity varying across different demographic groups. A comprehensive study conducted within a large Israeli healthcare organization reported an overall incidence of myocarditis of 2.13 cases per 100,000 vaccinated individuals within 42 days post-vaccination. The highest incidence was observed among males aged 16 to 29 years, reaching 10.69 cases per 100,000. Further analysis indicated that the risk of myocarditis was significantly elevated after the second dose of the vaccine, particularly in young males (193, 200). Clinical presentations of vaccine-associated myocarditis were predominantly mild to moderate, with symptoms typically manifesting within 3 to 5 days following the second vaccine dose. Most patients experienced chest pain and had elevated cardiac biomarkers, but severe outcomes were rare. In the Israeli cohort, only one case progressed to cardiogenic shock, and there was one death of unknown cause among patients with preexisting cardiac conditions (193). Long-term follow-up data suggest a favorable prognosis for individuals who developed myocarditis post-vaccination. In the aforementioned study, among patients who exhibited left ventricular dysfunction during hospitalization, subsequent evaluations showed normalization of cardiac function in the majority of cases over a median follow-up period of 83 days. These findings underscore the importance of continued surveillance and research to further elucidate the mechanisms, risk factors, and long-term outcomes associated with vaccine-related myocarditis, particularly in younger male populations.

#### 3.3.2 Dendritic cell-based vaccines

DC-based vaccines harness the natural ability of dendritic cells to present antigens and activate T cells. By loading dendritic cells with tumor antigens ex vivo and reintroducing them into the patient, these vaccines elicit a potent immune response targeted specifically at cancer cells (201). According to clinical studies, DC-based vaccinations are generally well tolerated, with side effects usually confined to minor injection site responses or flu-like symptoms. This approach avoids the systemic toxicities often associated with conventional cancer treatments, such as chemotherapy or radiation (202). DC vaccines represent a rapidly advancing class of cancer immunotherapies known for their personalized approach and excellent safety profile, particularly with regard to cardiovascular tolerability. Evidence from clinical trial NCT01280552 revealed that DCs pulsed with tumor-specific immunogenic peptides resulted in a longer median overall survival (18.3 months) compared to unpulsed DCs (16.7 months). The progression-free survival (PFS) was also notably improved in the antigen-pulsed group, emphasizing the therapeutic potential of this antigen-targeting strategy. However, outcomes are not universally favorable; for example, trial NCT02332889 reported no clinical benefit in a pediatric patient with relapsed high-grade glioma receiving a combination of autologous DC vaccination, poly-ICLC, and decitabine, highlighting the influence of tumor type and individual immune context on efficacy. Meanwhile, trial NCT01067287 is assessing the combined use of DC vaccines and PD-1 checkpoint inhibition following stem cell transplantation in multiple myeloma, though results remain pending. Despite these mixed efficacy outcomes, a consistent trend across studies is the excellent safety record of DC vaccines, particularly their negligible association with cardiotoxic complications. This differentiates them from therapies such as CAR T cells or ICIs, which have well-documented risks including myocarditis, cytokine release syndrome, and arrhythmias. To further boost their immunogenic potential, innovative approaches are being investigated. One such strategy involves transfecting DCs with mRNA encoding human telomerase reverse transcriptase (hTERT) to promote a potent cytotoxic T-cell response in prostate cancer (NCT01153113). Similarly, a more complex formulation using mRNA plus tumor cell lysates to load DCs has been explored in the treatment of acute myeloid leukemia (NCT00514189), allowing for a broader antigenic repertoire though at the cost of increased production complexity. Taken together, DC-based vaccines offer a promising and safe platform for cancer immunotherapy, particularly appealing for patients at risk of cardiovascular adverse events. Their ability to stimulate a tailored immune response with minimal systemic toxicity underscores their potential as a next-generation treatment strategy worthy of continued clinical exploration.

#### 3.3.3 DNA vaccines

DNA vaccines involve the direct delivery of genetic material encoding tumor-associated antigens into the patient’s cells, enabling *in situ* antigen expression and subsequent immune activation. These vaccines have shown significant safety advantages due to their non-replicating nature, reducing the risk of insertional mutagenesis or undesired effects on the genome. Furthermore, DNA vaccines are inherently non-infectious and do not involve live vectors, further enhancing their safety. In cancer therapy, DNA vaccines have demonstrated promising results in generating both humoral and cellular immune responses with minimal adverse events reported in clinical trials (203). Both dendritic cell vaccines and DNA vaccines exhibit favorable safety profiles with predominantly mild adverse events and low incidences of serious complications. DC vaccines, being cell-based therapies, involve complex manufacturing processes and logistical challenges but have demonstrated excellent tolerability in clinical settings. DNA vaccines offer advantages in production scalability and stability, with minimal adverse effects observed in trials. However, theoretical concerns such as the potential for anti-DNA antibody production exist, though not commonly observed in practice (**Table 8**).

**Table 8:** Comparative safety profile of dendritic cell vaccines and DNA Vaccines.

Feature	Dendritic Cell (DC) Vaccines	DNA Vaccines	Reference
Vaccine Platform	Autologous or allogeneic dendritic cells pulsed with tumor antigens	Plasmid DNA encoding tumor antigens	(273–283)
Mechanism of Action	Ex vivo antigen loading and maturation of dendritic cells, followed by reinfusion to stimulate T-cell responses	*In vivo* transfection of host cells leading to antigen expression and immune activation	
Common Adverse Events	Mild local reactions (e.g., injection site erythema), low-grade fever, fatigue	Mild local reactions, transient fatigue, rare systemic effects	
Serious Adverse Events (SAEs)	Rare; low incidence of Grade ≥3 adverse events	Rare; low incidence of Grade ≥3 adverse events	
Autoimmunity Risk	Low; minimal evidence of inducing autoimmunity	Low; theoretical risk due to potential for anti-DNA antibody production	
Long-term Safety Data	Favorable; low incidence of adverse events over extended follow-up periods	Favorable; long-term studies show minimal adverse effects	
Production Complexity	High; requires personalized cell processing and quality control	Moderate; scalable production with standardized protocols	
Storage and Stability	Limited shelf-life; requires cold chain logistics	High stability; can be stored at standard refrigeration or freezing temperatures	
Regulatory Status	Several DC vaccines have reached clinical trials; limited approvals	Multiple DNA vaccines in clinical trials; some approved for veterinary use	

DNA-based neoantigen vaccines have emerged as a prominent approach in cancer immunotherapy and currently rank alongside mRNA vaccines as one of the most commonly investigated platforms in ongoing clinical trials. Due to their strong inherent immunogenic properties, these vaccines typically do not require adjuvant support, yet they are often administered in conjunction with immune checkpoint inhibitors such as durvalumab or nivolumab to potentiate antitumor responses. For example, clinical studies NCT03199040 and NCT04397003 are evaluating patient outcomes by comparing DNA vaccination alone versus its combination with durvalumab. GNOS-PV02, a prototypical DNA vaccine, is being assessed in trial NCT04251117 alongside plasmid-encoded interleukin-12 and pembrolizumab, showcasing the platform’s adaptability for combinatory immunotherapy regimens. In addition to these combination approaches, DNA neoantigen vaccines are also being explored as stand-alone therapies. Trial NCT03122106 involves the use of a personalized DNA vaccine that integrates prioritized tumor neoantigens and mesothelin epitopes into a pING plasmid vector for patients following surgical resection and chemotherapy. Another example is trial NCT03988283, which investigates DNA vaccine monotherapy in pediatric populations with treatment-resistant or recurrent brain tumors. To optimize intracellular delivery, several of these trials utilize the TDS-IM system—a microneedle-based dermal delivery platform designed to facilitate efficient DNA transfection while minimizing systemic reactogenicity. One of the distinguishing advantages of DNA vaccines is their favorable safety profile, particularly concerning cardiovascular outcomes. Unlike CAR T-cell therapies and immune checkpoint inhibitors, which carry substantial risks such as myocarditis, cytokine release syndrome, and arrhythmogenic complications, DNA vaccines typically induce localized immune activation with minimal systemic inflammation. Additionally, their non-viral delivery mechanisms further reduce the likelihood of off-target immune effects. These characteristics make DNA-based neoantigen vaccines a compelling option within the immunotherapy landscape, especially for patients with preexisting cardiovascular conditions or those at heightened risk of cardiotoxicity.

#### 3.3.4 Neoantigen vaccines

Neoantigen vaccines represent a personalized cancer therapy that targets unique mutations present only in tumor cells. By leveraging advanced sequencing and bioinformatics, patient-specific neoantigens are identified and incorporated into vaccine formulations. The personalized nature of these vaccines ensures a high degree of tumor specificity, minimizing off-target effects. Clinical studies have shown that neoantigen vaccines are generally safe, with side effects primarily confined to mild injection site reactions or transient flu-like symptoms. This safety profile, combined with their potential to induce durable and specific anti-tumor immunity, positions neoantigen vaccines as a transformative approach in cancer immunotherapy (204). Recent early-phase clinical trials (NCT02950766) evaluating the safety of neoantigen vaccines have demonstrated a highly favorable safety profile. In one such study, the most common adverse events were low-grade, self-limited injection-site reactions, which occurred in 100% of patients, and transient flu-like symptoms, reported in 8 out of 9 patients (205). Importantly, no patient experienced a grade 3 or higher (dose-limiting) toxicity, underscoring the generally benign tolerability of this immunotherapy modality. These findings support the notion that neoantigen vaccines, which are designed to elicit precise and personalized immune responses against tumor-specific epitopes, are associated with minimal systemic toxicity and a low risk of serious immune-mediated complications, including cardiovascular adverse events. In the same clinical investigation (NCT03480152), the administration of mRNA-based neoantigen vaccines was also associated with gastrointestinal side effects (206), specifically mild episodes of nausea and vomiting. These symptoms were transient and non-severe, reflecting the overall tolerability of the vaccine formulation. Their occurrence highlights that while mRNA neoantigen vaccines are generally safe and well-tolerated, they can still elicit mild systemic reactions, likely due to innate immune activation or vaccine-related inflammatory responses.

### 3.5 Cytokine-based therapies

Although interferon alphas are mainly known for their neuropsychiatric and immunomodulatory effects, they have also been linked to a wide range of adverse reactions. These effects span multiple physiological systems, including the nervous and sensory systems, cardiovascular and respiratory functions, endocrine and metabolic pathways, hematologic profiles, urinary tract health, and skin conditions. Among the cardiovascular complications reported, pericarditis has emerged as a notable side effect, alongside other cardiovascular concerns. The occurrence of pericarditis and other related cardiovascular issues highlights the multifaceted nature of interferon alpha-induced toxicity, underscoring the importance of close monitoring in patients undergoing interferon-based therapies. Given the diverse range of potential adverse reactions, clinicians must remain vigilant in assessing the overall health of patients, particularly those with pre-existing conditions or those receiving long-term treatment, to mitigate the risks associated with these side effects. Further research into the mechanisms driving these systemic effects is essential to improve patient management and therapeutic outcomes (207–209). Additionally, once interferon was removed, cancer patients’ cardiomyopathy with left ventricular dilatation improved, allowing for the use of lower dosages of treatment (210). Pegylated interferon alfa-2b has been associated with serious cardiovascular complications, including acute myocardial infarction, pericarditis, and pericardial effusion resulting in cardiac tamponade. It has also been linked to the onset of sick sinus syndrome, a condition that can lead to significant arrhythmias. In one notably severe case, an orthotopic heart transplant recipient experienced allograft failure and ultimately passed away, with interferon-related toxicity identified as the underlying cause. These adverse events underscore the potential risks associated with pegylated interferon alfa-2b, particularly in individuals with pre-existing cardiovascular conditions or those who have undergone complex surgeries like heart transplants. The cardiovascular complications associated with this drug highlight the need for vigilant monitoring and careful management during treatment, as the severity of these side effects can significantly impact patient outcomes. Further investigation is necessary to elucidate the underlying mechanisms of interferon-induced toxicity, especially about its cardiovascular effects, to guide clinical decision-making and enhance patient safety (211–215). Both interferon alfa-2a and interferon alfa-2b have been implicated in the development of interstitial lung disease. However, the incidence is higher with interferon alfa-2a, whereas elevated doses of interferon alfa-2b are more frequently associated with this condition (216–221). Interferon gamma has been associated with cardiovascular complications, particularly at elevated doses, leading to conditions such as ventricular tachycardia, arrhythmias, hypotension, and coronary vasospasm (222, 223).

### 3.6 Monoclonal antibodies

There is still some misunderstanding in the medical literature about the exact meanings and consequences of the terms “cardiac hypersensitivity” and “cardiac toxicity,” especially when these terms are used to describe the immediate side effects of administering therapeutic monoclonal antibodies. Cardiac hypersensitivity refers to an immunologically mediated reaction, usually an immediate (Type I) or delayed (Type IV) hypersensitivity reaction that affects the heart. Unlike classic cardiotoxicity, cardiac hypersensitivity is not dose-dependent and may occur unpredictably, even at low or first-time exposure to a drug or vaccine (224). More broadly, cardiac toxicity usually refers to a cardiovascular side effect that depends on the treatment dosage and that persists and continues to show symptoms long after the administration of the causing medication has stopped. The development of a fibrotic response is an abnormal condition typically confirmed through histological analysis represents the most severe outcome of cardiac toxicity, though such confirmation has yet to be adequately performed or documented. However, when characterizing adverse events associated with therapeutic monoclonal antibodies, the term cardiac hypersensitivity may offer a more accurate and appropriate description than cardiac toxicity. For this reason, it is advised that this term be used instead of the other one. A hypersensitive reaction is characterized by an inflammatory reaction that is independent of the drug’s dosage, can occur at any stage of treatment, even when the drug is present in trace amounts, and is often accompanied by the development of anti-drug antibodies. These anti-drug antibodies are primarily IgG isotype, but it is important to note that a subgroup of hypersensitivity reactions can also involve IgE antibodies, which are known to elicit a different immune response; in fact, patients have been reported to experience IgE reactions specifically directed against therapeutic antibodies, especially those who have been treated with rituximab, a commonly used monoclonal antibody in clinical settings. To enhance patient care during monoclonal antibody therapy and promote clarity in medical discussions, it is essential to distinguish between these two terms. A clear and thorough understanding of their meanings can significantly impact both immunotherapy research and clinical practice. Within the context of monoclonal antibody treatment, it is vital for researchers and healthcare professionals to carefully evaluate these concepts to foster clearer communication and ultimately improve patient outcomes (225).

A novel class of anti-cancer treatments called antibody-drug conjugates (ADCs) is being used more and more to treat a variety of cancers, including hematologic and solid tumors. Frequently called “magic bullets,” these substances provide focused cancer therapy (226–228). ADC is made up of a monoclonal antibody (mAb), payload, and linker (229). ADCs are one of the most well-known fields in cancer medication research and development because of their exceptional capacity to combine accurate targeting with strong cytotoxic effects. This dual capacity makes it possible to eradicate cancer cells effectively and selectively (229). ADCs were developed because they offer improved targeting and higher cytotoxicity compared to standard cytotoxic chemotherapy, while also reducing the systemic toxicity that anti-tumor drugs often produce (230). Despite being a type of targeted chemotherapy, ADC usage has been linked to increased toxicity and unfavorable side effects in some cases (231). A patient’s quality of life and long-term survival may be significantly and potentially fatally impacted by adverse cardiovascular events (232). Cancer patients’ prognosis might be greatly impacted by adverse cardiovascular consequences. ADCs may be harmful to the cardiovascular system, according to evidence from the FAERS database. According to a recent study, there have been repeated instances of cardiotoxicity linked to ADCs that include trastuzumab deruxtecan (T-DXd) (233). The risk of ADCs developing into CVD is still up for debate, despite the fact that they are often prescribed and utilized in therapeutic settings. Although it hasn’t been verified yet, some research has shown a link between cardiotoxicity and trastuzumab deruxtecan and trastuzumab emtansine. Profoundly paraphrase in an intellectual manner (233, 234). Furthermore, it is yet unknown if additional ADCs pose a risk of cardiotoxicity.

Cardiovascular toxicity encompasses a range of conditions affecting the heart and blood vessels, including cardiomyopathy, heart failure, myocarditis, arrhythmias, coronary artery disease, early-onset valvular disorders, high blood pressure (hypertension), and the risk of thromboembolism. These conditions collectively represent the harmful effects of certain substances or diseases on cardiovascular health (235, 236). Previous research has explored the adverse events associated with ADCs using the FAERS database, both internationally and domestically. One study specifically analyzed adverse event signals for two ADCs, while another investigation concentrated on liver-related injuries caused by ADCs (234, 237). However, no study has used the FAERS database to look at the cardiovascular adverse events linked to ADCs. Therefore, by examining the cardiovascular adverse effects associated with ADCs, this study seeks to close that gap.

## 4 Future perspective

**Figure 5:**
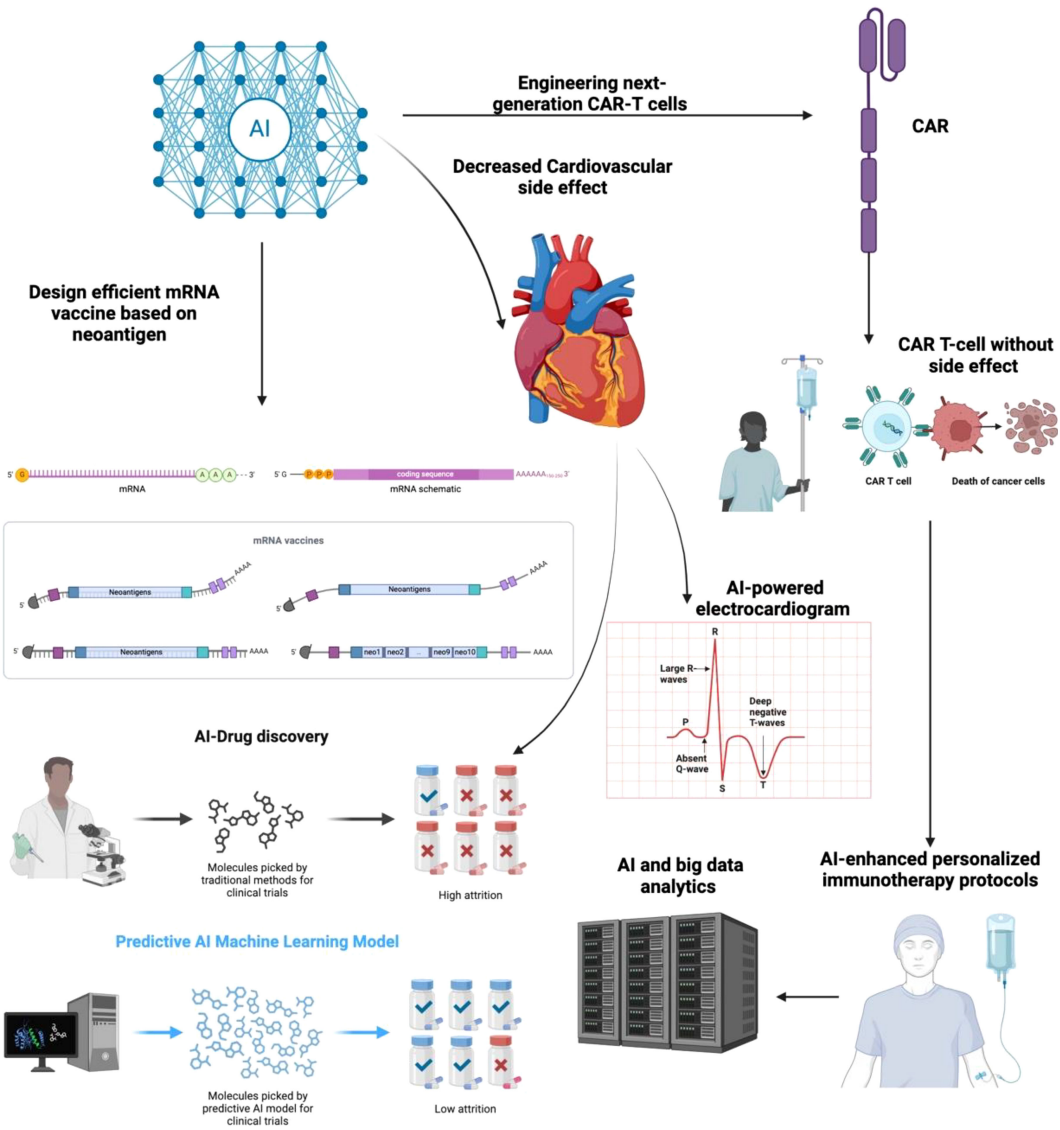
The integration of artificial intelligence (AI) in cancer immunotherapy for optimizing CAR-T cell therapy and mRNA vaccine design. AI-driven approaches facilitate the development of next-generation CAR-T cells with reduced cardiovascular side effects, enhance AI-powered electrocardiogram analysis, and improve drug discovery through predictive machine learning models. AI and big data analytics contribute to personalized immunotherapy protocols, while AI-based neoantigen prediction enables the design of efficient mRNA vaccines. This comprehensive AI-driven strategy enhances the safety and efficacy of immunotherapy in cancer treatment.

The future of immunotherapy holds immense potential to revolutionize cancer treatment by advancing precision medicine and mitigating adverse effects, including cardiovascular complications (**Figure 5**). As our understanding of the immune system’s complexities deepens, several promising avenues emerge (238, 239). Developing novel strategies to minimize immune-related cardiovascular toxicities, such as predictive biomarkers for early detection and risk stratification, will enhance patient safety and therapeutic outcomes.

Immunotherapy, particularly with ICIs and mAbs is revolutionizing cancer treatment and is now being utilized across a broader range of malignancies. Notably, ICIs have recently been approved for use in adjuvant and neoadjuvant settings for early-stage cancers, where long-term quality of life and the prevention of adverse events are of increasing importance. Despite fast-track regulatory approvals, the full spectrum of ICI-related toxicities both short- and long-term remains insufficiently understood, especially regarding their impact on patient-reported outcomes and quality of life. Some irAEs, particularly those involving the cardiovascular system, can significantly affect patient survival and necessitate treatment discontinuation. Approximately one-third of the 1.8 million new cancer cases diagnosed annually are expected to require complex immune-oncological therapies, which elevates the potential risk for serious irAEs. Several promising strategies are under investigation to mitigate immune-related cardiotoxicity. IL-6 inhibitors such as tocilizumab have shown efficacy in attenuating CRS without diminishing the therapeutic efficacy of CAR T-cell therapies. Additionally, JAK-STAT pathway inhibition via agents like ruxolitinib offers another cardioprotective avenue. In patients with ICI-related myocarditis, combination therapy with abatacept and ruxolitinib has been associated with a reduced incidence of major adverse cardiac events (MACE). Co-therapies that combine ICIs with cardioprotective agents may represent a future direction in balancing anti-tumor efficacy with cardiovascular safety. Given the unique nature of immunotherapy-induced cardiotoxicity, it is essential to develop tailored risk assessment models. In the future, validated predictive scores may facilitate the early identification of high-risk patients and guide clinical decision-making for immune-oncological therapies (240).

Furthermore, engineering next-generation CAR-T cells with reduced off-target effects and designing less toxic immune checkpoint inhibitors represent critical areas of innovation (241). Moreover, the development of advanced vaccine platforms, including mRNA vaccines and neoantigen-based vaccines, offers a safer and more targeted approach to cancer treatment, with the ability to elicit strong and durable anti-tumor immune responses. Innovations in cytokine engineering and delivery methods, such as localized cytokine release or nanoparticle-based systems, could significantly reduce systemic toxicity, including cardiovascular side effects. Future research must also address disparities in access to immunotherapy and its long-term effects on quality of life, particularly as survival rates improve. With a focus on improving safety profiles, tailoring treatments to individual patients, and expanding therapeutic options, immunotherapy is poised to reshape cancer care while minimizing its unintended consequences, including cardiovascular adverse events.

CAR T-cell therapy and ICIs have revolutionized cancer immunotherapy, yet they pose significant cardiovascular risks, including myocarditis, arrhythmias, hypertension, and thromboembolic events. The integration of artificial intelligence (AI) offers a transformative approach to predicting, preventing, and managing these complications, ultimately improving patient outcomes and expanding the safe application of these therapies. AI-driven predictive modeling, utilizing machine learning (ML) and deep learning (DL), can analyze vast datasets from clinical trials, patient records, multi-omics (genomics, transcriptomics, proteomics), and imaging studies to identify individuals at higher risk of cardiovascular toxicity. By integrating these data, AI can develop personalized risk stratification models, allowing clinicians to preemptively adjust treatment plans, incorporate cardioprotective strategies, and monitor high-risk patients more closely. Moreover, AI-powered electrocardiogram (ECG) and echocardiographic analysis can enhance early detection of cardiotoxicity by identifying subtle changes in cardiac function before symptoms manifest (242). Real-time AI-assisted monitoring systems, including wearable biosensors and remote patient monitoring platforms, can track heart rate variability, blood pressure fluctuations, and biomarkers of cardiac injury, providing continuous risk assessment and enabling early intervention. In addition to risk prediction, AI can aid in optimizing immunotherapy regimens to reduce cardiovascular complications. In silico modeling and AI-based drug discovery approaches can refine CAR T-cell designs, improving antigen specificity while minimizing inflammatory responses that contribute to myocardial damage. Similarly, AI can help identify novel biomarkers for immune-related myocarditis, guiding the development of targeted prophylactic and therapeutic strategies. As AI algorithms become more explainable and widely adopted in clinical practice, their role in cardiovascular risk management for immunotherapy patients will likely evolve into real-time decision support systems (243, 244). These systems will assist oncologists and cardiologists in dynamically adjusting therapy based on an individual’s evolving risk profile, ensuring a balance between maximizing therapeutic efficacy and minimizing adverse effects. Future research should focus on integrating AI-driven cardiotoxicity models into routine clinical workflows, enhancing collaboration between oncology and cardiology specialists, and developing AI-enhanced personalized immunotherapy protocols (245). With advancements in AI and big data analytics, the next decade will likely witness a paradigm shift where cardiovascular complications of CAR-T and ICI therapies are not just managed but proactively prevented, ensuring safer and more effective cancer treatment. Emerging evidence suggests that ICANS may contribute to cardiovascular complications through disruptions in the neuro-cardiac axis. Neuroinflammation associated with ICANS can impair autonomic regulation, leading to dysregulation of heart rate and blood pressure. For instance, a case study reported persistent orthostatic hypotension and tachycardia in a patient following CAR T-cell therapy, attributed to autonomic dysfunction secondary to ICANS (246). Furthermore, the intrinsic cardiac nervous system (ICNS), which plays a crucial role in cardiac autonomic control, may be affected by neuroinflammatory processes. Disruption of the ICNS can lead to arrhythmias and other cardiac dysfunctions (247). These findings underscore the importance of monitoring cardiovascular function in patients experiencing ICANS and suggest that interventions targeting autonomic dysfunction may be beneficial in mitigating associated cardiovascular risks.

## 5 Challenges and limitation

Despite the transformative impact of immunotherapy in oncology, its application is fraught with substantial challenges and limitations, particularly concerning cardiovascular complications. These challenges are multifaceted, spanning from unpredictable immune responses and heterogeneous patient outcomes to insufficient long-term safety data and inadequate risk stratification models. As immunotherapeutic agents especially ICIs, CAR T-cell therapy, and cancer vaccines gain clinical prominence, a critical evaluation of their limitations is essential for optimizing their use while minimizing harm (248).

### 5.1 ICIs: balancing efficacy and autoimmunity

One of the major challenges associated with ICIs is their propensity to trigger immune-related adverse events (irAEs), including cardiovascular toxicities such as myocarditis, pericarditis, arrhythmias, and heart failure. These adverse events are often life-threatening and difficult to predict. While ICIs such as anti-PD-1, anti-PD-L1, and anti-CTLA-4 have shown remarkable success in prolonging survival in cancers like melanoma and lung cancer, they simultaneously disrupt immune homeostasis, leading to unintended immune activation against healthy tissues. Cardiovascular irAEs, though less frequent than dermatologic or gastrointestinal ones, carry high morbidity and mortality rates, with myocarditis alone exhibiting a case fatality rate of 25–50%.

Furthermore, combination therapies, such as anti-PD-1 plus anti-CTLA-4, exacerbate this issue, significantly increasing the risk and severity of irAEs. For example, myocarditis risk is nearly five times higher in combination therapy compared to monotherapy. Yet, early clinical trials were not adequately powered to detect these rare but severe events, leading to a gap in post-marketing surveillance and delayed recognition of cardiovascular toxicity profiles. Additionally, patients with pre-existing autoimmune diseases or cardiovascular conditions are often excluded from trials, limiting the generalizability of findings to real-world settings. Another limitation is the absence of reliable biomarkers to predict which patients will develop cardiovascular complications. Although research has suggested that elevated levels of troponin or NT-proBNP may indicate early myocardial injury, these markers are nonspecific and can be confounded by cancer-related stress or comorbidities. There is an urgent need for validated, immunotherapy-specific biomarkers to guide clinical decision-making and facilitate early intervention.

### 5.2 CAR T-cell therapy: potent but perilous

CAR T-cell therapy, particularly in hematologic malignancies, represents a revolutionary advance in personalized medicine. However, its utility is limited by its unique and severe toxicity profile, including CRS and ICANS. Cardiovascular toxicity, while less studied, is an underappreciated yet serious complication. CRS-induced systemic inflammation can lead to vasodilation, capillary leak syndrome, and myocardial depression, culminating in arrhythmias, hypotension, and even cardiogenic shock. A critical challenge lies in the heterogeneity of cardiovascular events and their temporal relationship with therapy. Unlike chemotherapy-induced cardiotoxicity, which tends to be dose-dependent and cumulative, CAR T-cell-associated cardiotoxicity is acute, unpredictable, and may occur in the absence of pre-existing heart disease. Moreover, the severity of CRS is not always correlated with the degree of cardiac injury, complicating monitoring and treatment efforts.

There is also a lack of standardized protocols for cardiac monitoring in CAR T-cell recipients. While some centers perform baseline echocardiograms and biomarker assessments, there is no consensus on the frequency or duration of follow-up. This heterogeneity in practice may lead to missed diagnoses and delayed management of cardiotoxic events. Furthermore, current interventions (e.g., corticosteroids or tocilizumab) are designed to suppress immune activation broadly and may reduce the efficacy of CAR T cells, underscoring the need for selective immunomodulation strategies. Another significant limitation is the exclusion of patients with impaired cardiac function from CAR T-cell trials, which creates uncertainty about the safety and efficacy of this therapy in populations most vulnerable to cardiovascular complications.

### 5.3 Cancer vaccines: a promise tempered by uncertainty

Cancer vaccines, including mRNA-based and dendritic cell (DC)-based platforms, offer a promising route to induce tumor-specific immunity. However, their cardiovascular safety profiles remain poorly characterized. mRNA vaccines, such as those developed for SARS-CoV-2, have been linked to myocarditis and pericarditis, particularly in younger males. While these findings have raised concerns about the broader application of mRNA platforms in oncology, there is currently insufficient data on their cardiovascular effects in cancer patients, who often present with multiple comorbidities and altered immune responses.

The challenges extend to the potential for unintended systemic immune activation. Unlike conventional vaccines, therapeutic cancer vaccines are designed to stimulate a potent cellular immune response, which may inadvertently target cardiac tissues if cross-reactive antigens are present. This risk is heightened in neoantigen-based vaccines, where each formulation is unique and personalized, complicating safety monitoring and regulatory oversight.

Moreover, logistical challenges, such as the time required to develop personalized vaccines, limit their application in rapidly progressing cancers. The integration of cardiovascular screening into vaccine trials is also not routine, further limiting our understanding of vaccine-induced cardiotoxicity.

### 5.4 Cytokine-based therapies and monoclonal antibodies: double-edged swords

Cytokine-based therapies, including interferons and interleukins, are associated with a broad range of adverse effects across various organ systems, with cardiovascular toxicity being particularly concerning. Interferon-alpha, for instance, has been linked to pericarditis, myocardial infarction, arrhythmias, and cardiomyopathy. These effects are thought to stem from immune-mediated endothelial injury and inflammation but remain poorly defined mechanistically. High inter-individual variability in response to cytokine therapies further complicates risk assessment and management.

Similarly, monoclonal antibodies and ADCs have shown promise in targeting tumors with precision. However, they can induce “cardiac hypersensitivity” reactions that are independent of dosage and may involve IgE-mediated mechanisms, especially in the case of rituximab. This unpredictability complicates therapeutic planning and necessitates careful pre-treatment screening. The delayed onset of cardiotoxicity, sometimes occurring months after therapy, adds another layer of complexity to surveillance and diagnosis.

### 5.5 Gaps in real-world evidence and long-term safety

Another significant challenge is the scarcity of long-term safety data. Most clinical trials have short follow-up periods, insufficient for assessing chronic cardiovascular outcomes such as atherosclerosis progression, valvular disease, or delayed cardiomyopathy. Additionally, real-world studies are limited by underreporting and variability in documentation of cardiovascular events, especially in community oncology settings with limited access to cardio-oncology services.

Data on vulnerable populations, including the elderly, those with pre-existing cardiovascular disease, and racial or ethnic minorities, are also lacking. These groups may experience differential risks due to pharmacogenomic variations or baseline health disparities but remain understudied in clinical research.

### 5.6 Practical and ethical barriers

Finally, logistical and ethical constraints hinder the advancement of cardio-oncology as a subspecialty. There is a pressing need for interdisciplinary collaboration between oncologists and cardiologists, but institutional silos often impede communication. Resource limitations in low- and middle-income countries further exacerbate disparities in access to cardio-oncology care, limiting the global applicability of immunotherapy.

Ethically, the risk-benefit calculus becomes challenging when potentially life-saving immunotherapies carry a non-negligible risk of fatal cardiovascular events. Patients must be thoroughly counseled about these risks, yet this is complicated by the lack of robust predictive tools and variable physician expertise.

### 5.7 Limitations of preclinical models

Despite their indispensable role in elucidating mechanistic insights, preclinical models used to evaluate ICI-induced cardiotoxicity have notable limitations that restrict their translational relevance. Knockout mouse models, while useful for dissecting molecular pathways, fail to replicate the pharmacokinetic profiles of immune checkpoint inhibitors and may inadequately reflect the clinical disease context (249). Additionally, ICI administration in animal studies often employs murine-specific antibodies at supra-therapeutic doses that do not accurately mimic human dosing regimens. This may lead to either an overestimation or underrepresentation of immune-mediated cardiotoxic effects, limiting the applicability of findings to patient care. Another significant challenge is the inadequate representation of the human microbiome and environmental pathogen exposure in current animal models. These factors are increasingly recognized as critical modulators of both the efficacy and toxicity of ICI therapy. Furthermore, murine models inherently lack the capacity to reflect the full spectrum of human biological heterogeneity, including variations in age, sex, genetic background, comorbidities, and socio-environmental influences. As a result, findings derived from these models may not fully capture the complexity of ICI responses observed in diverse patient populations. Addressing these limitations requires the development of more sophisticated and human-relevant experimental systems, such as humanized mouse models or organ-on-chip platforms, to improve predictive accuracy and clinical translatability.

## 6 Conclusion

The advent of immunotherapy has significantly transformed cancer treatment, offering new hope for patients with previously limited therapeutic options. However, its widespread implementation has also highlighted a concerning spectrum of cardiovascular toxicities associated with immune checkpoint inhibitors, CAR T-cell therapy, and cancer vaccines. Myocarditis, arrhythmias, hypertension, heart failure, and thromboembolic events are among the most frequently reported cardiovascular adverse effects, posing substantial risks to patient health and treatment outcomes. The mechanisms underlying these toxicities are complex, often involving immune-mediated inflammation, cytokine release, and endothelial dysfunction. Given the increasing integration of immunotherapy into clinical practice, a multidisciplinary approach involving oncologists, cardiologists, and immunologists is essential for early identification, monitoring, and management of these complications. Strategies such as pre-treatment cardiovascular screening, biomarker-based risk assessment, and the use of cardioprotective agents may help mitigate these adverse effects while preserving the therapeutic benefits of immunotherapy. Future research should focus on optimizing immunotherapeutic approaches to minimize off-target effects and developing predictive models for cardiovascular toxicity. Advances in artificial intelligence, precision medicine, and biomarker discovery may pave the way for personalized treatment strategies that balance efficacy with safety. By addressing these challenges, the field of cardio-oncology can ensure that immunotherapy remains a viable and effective treatment option while safeguarding patient cardiovascular health.
